# Quantum Dots as a Good Carriers of Unsymmetrical Bisacridines for Modulating Cellular Uptake and the Biological Response in Lung and Colon Cancer Cells

**DOI:** 10.3390/nano11020462

**Published:** 2021-02-11

**Authors:** Joanna Pilch, Patrycja Kowalik, Piotr Bujak, Anna M. Nowicka, Ewa Augustin

**Affiliations:** 1 Faculty of Chemistry, Gdańsk University of Technology, Narutowicza Str. 11/12, 80-233 Gdańsk, Poland; 2 Faculty of Chemistry, University of Warsaw, Pasteura Str. 1, 02-093 Warsaw, Poland; pkowalik@ch.pw.edu.pl (P.K.); anowicka@chem.uw.edu.pl (A.M.N.); 3 Faculty of Chemistry, Warsaw University of Technology, Noakowskiego Str. 3, 00-664 Warsaw, Poland; piotrbujakchem@poczta.onet.pl

**Keywords:** unsymmetrical bisacridines, quantum dots, lung and colon cancer cells, cellular uptake, internalization, apoptosis, cellular senescence

## Abstract

Nanotechnology-based drug delivery provides a promising area for improving the efficacy of cancer treatments. Therefore, we investigate the potential of using quantum dots (QDs) as drug carriers for antitumor unsymmetrical bisacridine derivatives (UAs) to cancer cells. We examine the influence of QD–UA hybrids on the cellular uptake, internalization (Confocal Laser Scanning Microscope), and the biological response (flow cytometry and light microscopy) in lung H460 and colon HCT116 cancer cells. We show the time-dependent cellular uptake of QD–UA hybrids, which were more efficiently retained inside the cells compared to UAs alone, especially in H460 cells, which could be due to multiple endocytosis pathways. In contrast, in HCT116 cells, the hybrids were taken up only by one endocytosis mechanism. Both UAs and their hybrids induced apoptosis in H460 and HCT116 cells (to a greater extent in H460). Cells which did not die underwent senescence more efficiently following QDs–UAs treatment, compared to UAs alone. Cellular senescence was not observed in HCT116 cells following treatment with both UAs and their hybrids. Importantly, QD_green/red_ themselves did not provoke toxic responses in cancer or normal cells. In conclusion, QDs are good candidates for targeted UA delivery carriers to cancer cells while protecting normal cells from toxic drug activities.

## 1. Introduction

At present, cancer is one of the most serious diseases in the world. Despite the development of new cancer therapies, including immunotherapy and photothermal therapy, traditional chemotherapy is still the main method for cancer treatment [1]. However, effective methods of cancer therapy using conventional chemotherapeutic agents are still limited [2,3]. The most significant difficulties in conventional chemotherapy are poor bio-distribution to tumors, leading to toxic side effects in normal tissues and cells [4], as well as low drug solubility, the development of multiple drug resistances, and high dose requirements [5]. These issues can be potentially overcome through the delivery of anticancer drugs by nanoparticles (NPs) [6,7]. NP-based drug delivery can improve classical therapeutic strategies against cancer by increasing the bioavailability, pharmacokinetic characteristics, and accumulation of the active therapeutic agents at tumor sites [8], as well as decreasing their toxicity towards normal tissues [9,10]. Therefore, the delivery of drugs using NPs has become an increasingly important approach to antitumor therapy [11]. The most extensively explored nanocarriers used in target-specific drug delivery systems include liposomes, dendrimers, micelles, and nanoparticles [12,13]. The essential advantage of effective NP-based drug delivery systems is the enhanced internalization of nanocarriers to target cells (e.g., cancer cells), thus causing a reduction in toxicity of normal cells [14].

In our previous studies, we have applied quaternary quantum dots (QDs) as a drug nanocarrier. QDs are spherical semiconductor NPs with versatile surface chemistry, which can be modulated. They have high potential in various biological applications, including bioimaging and drug delivery systems [15,16]. Furthermore, QDs have many superiorities compared to other drug carriers, such as larger specific surface area, smaller size, and stronger adsorption capacity [17]. QD-based drug delivery provides a promising area for improving the efficacy of drugs and developing new therapies [18]. Unsymmetrical bisacridine derivatives (UAs), new anticancer compounds synthesized in our laboratory, were non-covalently attached to non-toxic QDs (Ag–In–Zn–S nanocrystals). These compounds exhibited high cytotoxic activity—principally against human pancreatic, lung, and colon cancer cells—as well as demonstrated high antitumor activity, preferentially against pancreatic cancer xenografts of human origin [19,20,21].

It has been demonstrated previously that an efficient procedure for the synthesis of QD−UA hybrids successfully increases the cytotoxic activity of UAs in cancer H460 cells and have protective effects on normal MRC-5 and CCD 841 CoN cells [22]. In the present study, we investigate whether the application of QDs as delivering UAs to cancer cells influences the cellular uptake and the biological response of lung H460 and colon HCT116 cancer cells, as well as normal MRC-5 and CCD 841 CoN cells. The cellular uptake kinetics and the mechanism of internalization of QDs–UAs were explored, as well as the influence of the linkage of UAs with QDs on the cell cycle progression, induction of apoptosis, and cellular senescence in cancer and normal cells.

## 2. Materials and Methods

### 2.1. Materials

Unsymmetrical bisacridine derivatives (UAs: C-2028 and C-2045) were synthesized at Gdańsk University of Technology, Department of Pharmaceutical Technology and Biochemistry. Quantum dots (QDs: QD_green_ and QD_red_) were synthesized at Warsaw University of Technology, and QD-UA hybrids were created at the University of Warsaw. Cytochalasin D, Amiloride, Dynasore, Pitstop 2, Filipin III, X-gal (5-bromo-4-chloro-3-indolyl-β-d-galactosidase), streptomycin, and penicillin were purchased from Sigma-Aldrich, St. Louis, MO, USA. All reagents and chemicals used in experiments were of analytical grade and were used as received.

#### Synthesis of QDs-UAs Hybrid

The procedure of the synthesis of QD–UA hybrids is described in detail in our previous paper [22] concerning the physicochemical properties of these hybrids. Briefly, unsymmetrical bisacridine derivatives were anchored at the QD surface via non-covalent interactions. The mixture of the component QDs modified with 11-mercaptoundecanoic acid (MUA) and UAs in the mass ratio 1:3 was stirred overnight at room temperature and then dialyzed (two times) against 0.02 M phosphate buffer pH 7.4 to remove unbound bisacridine derivative. The physical properties like size, zeta potential, fluorescence activity of the synthesized QD-UA hybrids were analyzed with the application of dynamic light scattering (DLS), zeta potential (ZP), transmission electron microscopy (TEM), and fluorescence measurements. 

**Figure 1:**
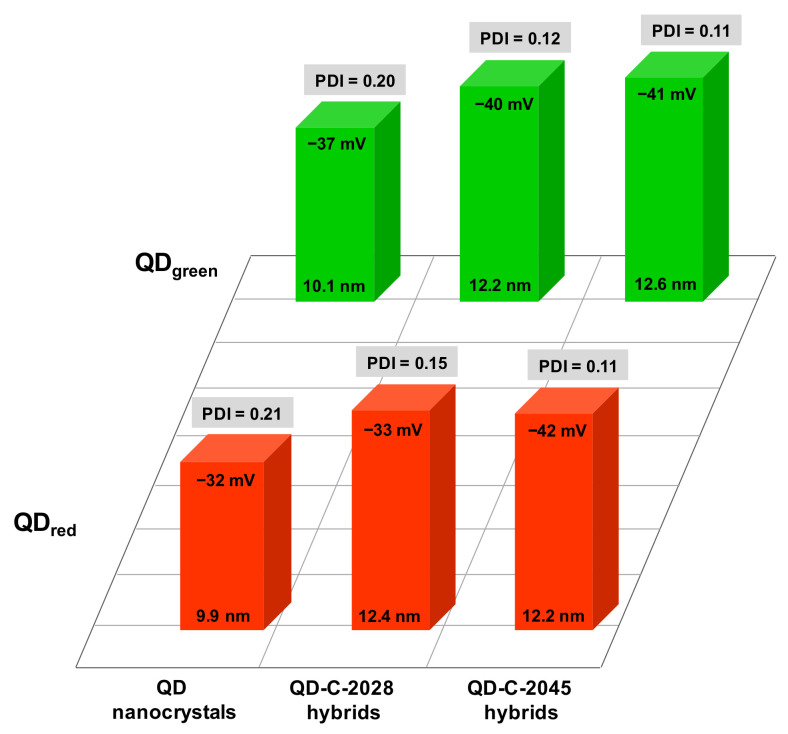
Mean size, polydispersity index (PDI), and zeta potential of quantum dot (QD) nanocrystals, QD−UA (unsymmetrical bisacridine derivatives) hybrids dispersed in PBS buffer based on dynamic light scattering (DLS) studies (*n* = 5).

It is known that efficient nanocarriers require the preparation of stable monodisperse populations of nanocarriers of a certain size [23]. The hydrodynamic diameter of the applied QD nanocrystals slightly increases (*ca.* 2 nm) after conjugation process with UAs, since the polydispersity index (PDI) significantly decreases, see Figure 1. A reduction in the PDI testifies that the size distribution of nanoconjugates is narrowing. The PDI values smaller than 0.1 are characterized by highly monodisperse nanocrystals. An excellent parameter characterizing the stability of the synthesized QD−UA hybrids is zeta potential (ZP), which is a measure of the magnitude of the electrostatic or charge repulsion/attraction between particles and is one of the fundamental parameters known to affect stability. The higher the value of ZP (positive or negative), the more stable the dispersion is; normally, a value of >30 mV indicates good stability. The dispersion of QD−UA hybrids under physiological conditions (PBS buffer, pH 7.4) was characterized by very good stability, since the values of ZP for all synthesized hybrids were significantly higher than −30 mV (Figure 1).

**Figure 2:**
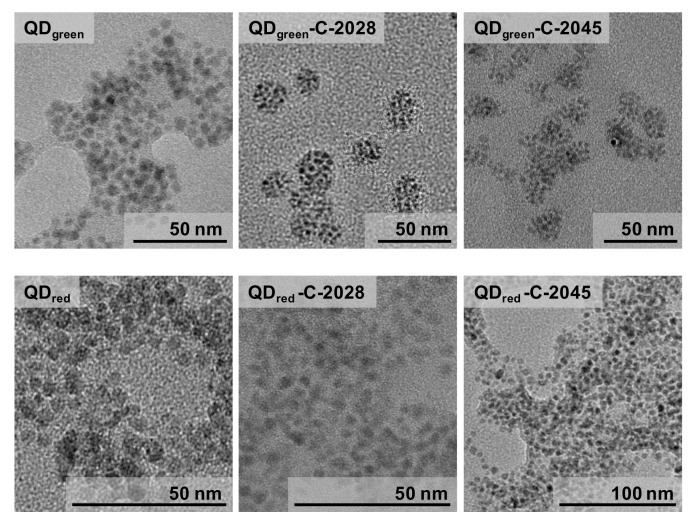
TEM images of QDs and their hybrids with UA compounds.

The successful conjugation of QDs with unsymmetrical bisacridine derivatives was also confirmed using electron microscopy. The obtained TEM images are presented in Figure 2. The TEM images showed that the diameter of hybrids slightly increases after the conjugation process, while the tendency for the aggregation decreases.

To confirm the successful anchoring of UA compounds at the QD surface, the fluorescence measurements were performed, since both applied quantum dots and unsymmetrical bisacridine derivatives are fluorescent active. The fluorescence spectra of QD_green_, QD_red_, C-2028, and C-2045 compounds alone (dashed lines and curves in insets) and their hybrids were shown in Figure S1 in the Supplementary Materials; SM. The conjugation of quantum dots with UA compounds led to the quenching of MUA-capped quantum dots’ fluorescence since the fluorescence of UAs had been significantly increasing. An increase in the UAs fluorescence confirmed that the amount of the drug transported to the cancer cells is in the form of hybrids with quantum dots and is higher than the free drug.

### 2.2. The Used Concentration of UAs, QDs, and QD−UA Hybrids

The cytotoxic activity of QDs, UAs, and QD−UA against H460, HCT116, MRC-5, and CCD 841 CoN cells was examined by MTT assay, described previously [22]. All experiments were performed in the concentrations of the QDs, UAs, and QD−UA, which corresponded to the estimated IC_80_ value for UAs alone following 72 h of incubation. The IC_80_ value for C-2028 and QD-C-2028 hybrids was 0.035 µM for all cell lines, for C-2045 and QDs-C 2045: 0.273 µM and 0.254 µM for H460/MRC-5 cells and HCT116/CCD 841 CoN cells, respectively. The concentration of unbound QD_green/red_, corresponding to the IC_80_ value of UAs alone in the hybrids (QDs−UAs) and was 0.0009 mg·mL^−1^ in all cell lines.

### 2.3. Cell Culture

Human cancer and normal cell lines were purchased from the American Type Culture Collection (ATCC; Manassas, VA, USA) and were tested negatively for mycoplasma using Universal Mycoplasma Detection Kit—ATCC-30-1012 K (ATCC). H460 cells (non-small cell lung carcinoma) were cultured in RPMI 1640 medium (Sigma-Aldrich, St. Louis, MO, USA). HCT116 cells (colorectal carcinoma) were cultured in McCoy’s 5A medium (Sigma-Aldrich, St. Louis, MO, USA). Both media contained: 10% fetal bovine serum (FBS; Biowest, Riverside, MO, USA), 100 µg·mL^−1^ of streptomycin, and 100 unit·mL^−1^ of penicillin. MRC-5 and CCD841CoN normal cells were cultured in EMEM medium (Eagle’s Minimal Essential Medium, Sigma-Aldrich, St. Louis, MO, USA). supplemented with 10% FBS, without antibiotics. All cells were incubated in a humidified atmosphere containing 5% CO_2_ at 37 °C. Experiments were performed with cells in the exponential phase of growth.

### 2.4. Applied Characterization Methods

Dynamic light scattering (DLS) and zeta potential (ZP) analysis were performed with a Zetasizer nano series apparatus (Malvern Panalytical Ltd., Malvern, UK) with a He-Ne (4 mW) laser at 632.8 nm. The experiments were carried out in PBS buffer at 25 °C, at least five times, with three freshly prepared samples. Transmission electron microscopy (TEM) measurements were performed on a Zeiss Libra 120 electron microscope (Oberkochen, Germany) operating at 120 kV. The elemental analysis was carried out with a multichannel Quantax 400 EDS system (Mannheim, Germany) with 125 eV xFlash Detector 5010, Bruker using 15 kV electron beam energy. Fluorometric measurements were performed using Scinco-FS2 spectrofluorometer (Scinco, Seoul, South Korea) with low volume (200 µL) optical cuvette (Product no. 16.100 F/Q/10 from Starna Scientific, Co, Ilford, UK) compatible in terms of size and aperture position with the apparatus.

### 2.5. Confocal Microscopy Imaging

Due to the fluorescence properties of QDs and UAs, they served as an intrinsic fluorescence probe to efficiently explore their cellular uptake. Briefly, 10^6^ cells were seeded in the 60 mm plate with glass coverslips (except control and QDs for 48 and 72 h—5 × 10^5^ cells were seeded) and incubated overnight. Next, cells were treated with QDs, UAs, and QD−UA hybrids at IC_80_ value for 1, 24, 48, and 72 h (and extra time point 6 h for H460 cells) of incubation. Moreover, to explore the mechanism of internalization of QD−UAs, cells were first preincubated with different inhibitors (at concentrations that were non-toxic to the cells): drug-free medium (no inhibitor), at 4 °C, 5 µM Cytochalasin D, 30 µM Amiloride, 80 µM Dynasore, 25 µM Pitstop 2 and 1.5 µM Filipin III for 30 min, followed by further incubation with QD_green_-C-2028 for 4 h. Furthermore, cells were washed twice in cold PBS and immediately observed using the CLSM (63× magnification; ZEISS LSM T-PMT, Magdeburg, Germany). The imaging conditions were: QD_green_ (excitation 300 nm, emission 543 nm), QD_red_ (excitation 663 nm, emission 691 nm), and UAs (excitation 528 nm, emission 553 nm). The Mean Fluorescence Intensity (MFI) of QDs, UAs, and QD−UA hybrids from the images was performed with ImageJ software (version 1.8.0., Wisconsin, Madison, USA).

### 2.6. Cell Cycle Analysis 

For DNA content analysis, the PI/RNase Staining Kit (BD Pharmingen, Franklin Lakes, NJ, USA) was used according to the manufacturer’s instruction. In short, cancer cells were seeded at a concentration of 10^6^ cells/100 mm plate (except control (untreated cells), QD_red_ and QD_green_—10^5^ and 10^4^ cells/plate for 72 and 144 h of incubation, respectively) and were allowed to adhere overnight. Then, cells were treated with QDs, UAs, and QD−UA hybrids for 24, 72, and 144 h at IC_80_ value. Cells were collected from plates by trypsinization, washed twice with PBS, fixed in ice-cold 80% ethanol, and left at −20 °C overnight. Next, the cells were centrifuged at 1500 rpm for 5 min at 4 °C and washed twice with PBS. Then, the cells were stained with 500 µL PI/RNase staining buffer at room temperature (RT) in the dark for 15 min and analyzed by flow cytometry (Accuri™ C6; Becton Dickinson, Franklin Lakes, NJ, USA). The data were analyzed using BD Accuri^TM^ C6 Software Version 1.0.264.21. Cell cycle analysis was performed at least three times.

### 2.7. Annexin V/Propidium Iodide (PI) Binding Assay 

To detect apoptosis, cells were stained using the FITC Annexin V Apoptosis Detection Kit I (BD Pharmingen, USA) according to the manufacturer’s instruction. Briefly, cancer cells following treatment with QDs, UAs, and QD−UA hybrids at IC_80_ value for 24, 72, and 144 h were harvested by trypsinization from plates, centrifuged at 1000 rpm for 5 min at 4 °C, and washed twice with cold PBS. Next, the cells were resuspended in 100 μL 1× binding buffer with 5 μL FITC Annexin V and 5 μL propidium iodide (PI) and incubated at RT in the dark for 15 min. Then, 400 μL 1× binding buffer was added to the cells and analyzed by flow cytometry (Accuri™ C6; Becton Dickinson, San Jose, CA, USA). The data were analyzed using BD Accuri^TM^ C6 Software Version 1.0.264.21 (San Jose, CA, USA). The analysis was carried out at least three times.

### 2.8. Changes of Mitochondrial Membrane Potential (ΔΨ_m_) 

Changes of ΔΨ_m_ in cells were performed using the Mitochondrial Membrane Potential Detection JC-1 Kit (BD Pharmingen, San Diego, CA, USA) according to the manufacturer’s instruction. In short, following incubation with QDs, UAs, and QD−UA hybrids at IC_80_ value for 24, 72, and 144 h, cancer cells were collected and centrifuged at 400 g for 5 min at 23 °C. Next, the cells were stained with 500 µL working solution (contained 5 μL JC-1 dye and 495 μL 1× assay buffer) and incubated at 37 °C in a CO_2_ incubator for 15 min. Afterward, the cells were washed twice with 1× assay buffer (2 mL and 1 mL, respectively), resuspended with 450 μL 1× assay buffer, and analyzed by flow cytometry (Accuri™ C6; Becton Dickinson, San Jose, CA, USA). The data were analyzed using BD Accuri^TM^ C6 Software Version 1.0.264.21 (San Jose, CA, USA). Each experiment was repeated at least three times.

### 2.9. Time-Lapse Recording 

H460 and HCT116 cancer cells (10^4^ per well) were seeded in 24-well plates (Cell Imaging Plate, Eppendorf). After 24 h of preincubation medium was replaced and QDs, UAs, and QD−UA hybrids were added into wells at IC_80_ value. Frames were acquired by Phase-Contrast microscopy (20× magnification, Olympus IX83, Tokyo, Japan) every 10 min for 72 h. These images were processed using the CellSens software.

### 2.10. Senescence-Associated β-Galactosidase Activity Assay 

For determination of cellular senescence, pH 6.0-dependent β-galactosidase expression was used as a marker. H460 and HCT116 cells were seeded at a 60 mm plate with coverslips at a density of 6 × 10^5^ cells/plate (except control (untreated cells), QD_red_, and QD_green_—3 × 10^4^ and 2 × 10^4^ cells/plate for 120 and 144 h of incubation, respectively) and incubated overnight. Following treatment with QDs, UAs, and QD−UA hybrids at IC_80_ value for 72, 120, and 144 h of incubation cells were washed three times with PBS and then Fixative Solution (0.2% glutaraldehyde and 2% formaldehyde in PBS) was added for 5 min. Next, the cells were washed three times with PBS again and stained with a Staining Solution containing 1 mg·mL^−1^ X-gal (5-bromo-4-chloro-3-indolyl-β-d-galactosidase). Following 12–16 h incubation at 37 °C, the cells were washed twice with PBS and observed using the light microscope (Olympus BX60, Tokyo, Japan) with the Nomarski interference contrast.

### 2.11. Statistical Analysis 

Data were expressed as a mean with standard deviation (SD) and collected from at least three independent experiments. Statistical analysis was performed by the Student’s *t*-test, and the differences of *p* < 0.05 between the two groups were considered as statistically significant: * *p* < 0.05, ** *p* < 0.01, *** *p* < 0.001.

## 3. Results

### 3.1. Cellular Uptake

**Figure 3:**
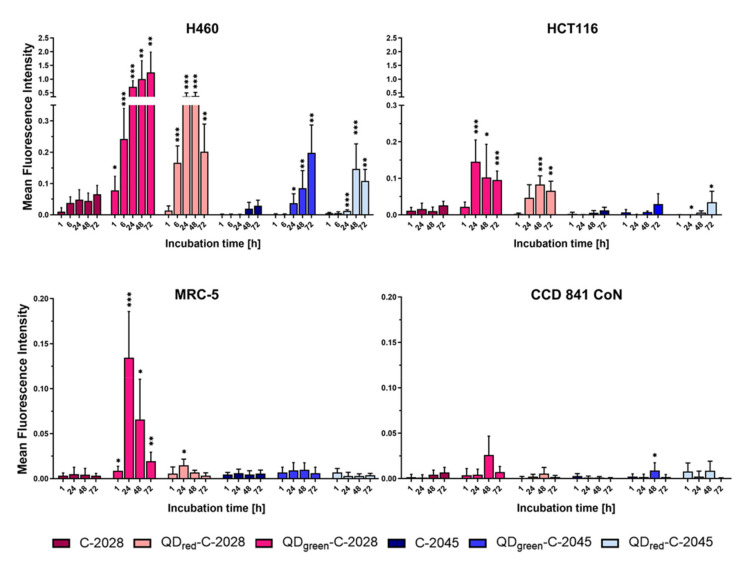
Cellular uptake of UAs, and QD−UA hybrids to cancer (H460 and HCT116) and normal (MRC-5 and CCD 841 CoN) cells for the time indicated and analyzed by Confocal Laser Scanning Microscope (CLSM). Mean Fluorescence Intensity (MFI) values of UAs and QDs−UAs were determined using ImageJ software. Data are expressed as the mean ± standard deviation of three independent experiments. * *p* < 0.05, ** *p* < 0.01, *** *p* < 0.001—statistically significant differences between the MFI of UAs in the cells incubated with UAs alone and QD−UA hybrids (Student’s *t*-test).

The cellular uptake of QDs, UAs, and QD−UA hybrids was investigated in cancer H460 and HCT116 cells, as well as in normal MRC-5 and CCD 841 CoN cells, using a Confocal Laser Scanning Microscope (CLSM) based on the fluorescence properties of these compounds (QDs and UAs). As shown in Figure 3 and Figure S2a–d (in the SM), the fluorescence intensity of free UAs in the cells was observed, starting with 1 h of incubation. The most intensive signals from UAs were detected in the case of C-2028 in H460 cells.

The fluorescence intensity of QD_green/red_ (QDs) was weak and did not change with the prolongation of incubation in all cell lines. UAs non-covalently attached to QDs (named QD−UA hybrids) significantly increased the amount of compound delivered to the cells, compared to UAs alone; especially for QD–C-2028 hybrids in H460 cells. Moreover, in these cells, the number of QD−UA hybrids increased over the time of incubation. This effect was stronger in the case of QD_green_–UA hybrids than for QD_red_−UAs. On the other hand, the fluorescence intensity of QDs−UAs in HCT116 cells was weaker than in H460 cells, but still higher than that of UAs alone. In MRC-5 cells, the highest fluorescence intensity of QD−UA hybrids was observed after 24 h of incubation, especially for QD_green_-UAs, which decreased following longer incubation times (up to 72 h). In CCD 841 CoN cells, the signals from both UAs alone and QD−UA hybrids were very weak, at the limit of the detection. However, the fluorescence intensity of signals from UAs alone and QD−UA hybrids was much weaker in normal cells compared to cancer cells.

### 3.2. Mechanism of Internalization

As QD−UA hybrids were delivered to the cells with different efficiencies, the next step of our study was to investigate the mechanism of their internalization. To explore this mechanism, we employed different pharmacological inhibitors of different pathways of endocytosis in non-toxic concentration, including Cytochalasin D (inhibition of macropinocytosis/phagocytosis, an inhibitor of actin polymerization), Amiloride (inhibition of macropinocytosis—MP, an inhibitor of Na^+^/H^+^ exchange), Dynasore (inhibition of clathrin-mediated endocytosis—CME, an inhibitor of the GTPase activity of dynamin), Pitstop 2 (inhibition of CME, an inhibitor of clathrin box ligands from binding to clathrin TD), Filipin III (inhibition of caveolae-mediated endocytosis—CavME, a sterol-binding agent causing the disassembly of caveolae), and a temperature of 4 °C (an inhibitor of the energy-dependent process) [24]. H460, HCT116, and MRC-5 cells were exposed to these inhibitors for 30 min and then incubated with QDs hybrids for 4 h. As the intensity of fluorescence signals in cells was the highest in the case of QD_green_–C-2028, this hybrid was selected for the internalization mechanism study. Moreover, the fluorescent signals of QD−UA hybrids in normal CCD 841 CoN cells were at the limit of detectability and, so, this cell line was not tested.

**Figure 4:**
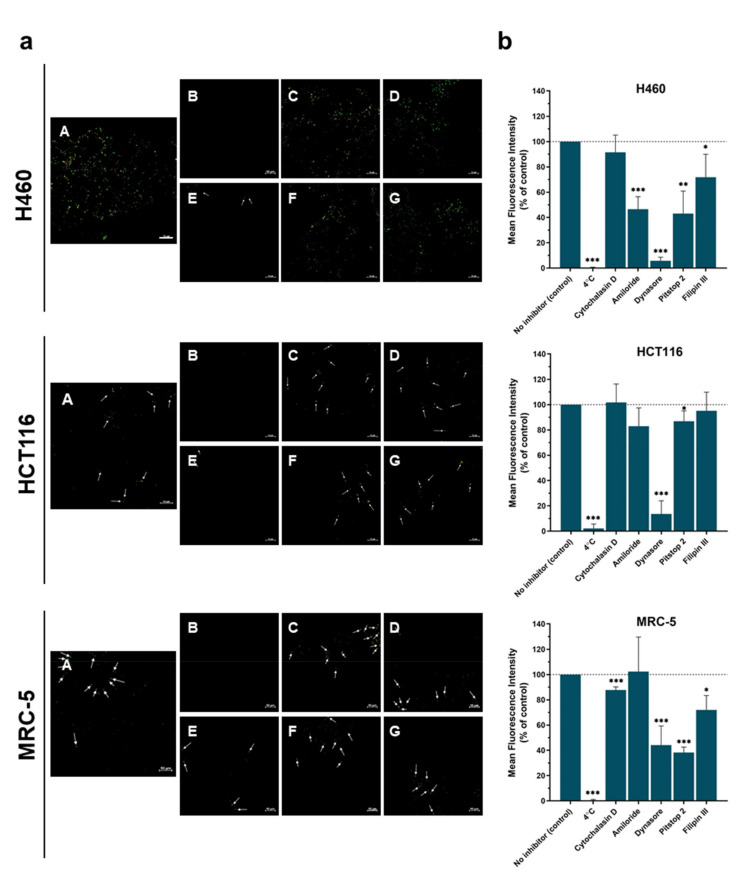
(**a**) The influence of different endocytosis inhibitors on the internalization of QD_green_-C-2028 in cancer and normal cells. H460, HCT116, and MRC-5 cells were preincubated with (**A**) drug-free medium (no inhibitor), (**B**) at 4 °C, (**C**) 5 µM Cytochalasin D, (**D**) 30 µM Amiloride, (**E**) 80 µM Dynasore, (**F**) 25 µM Pitstop 2, and (**G**) 1.5 µM Filipin III for 30 min, followed by further incubation with QD_green_-C-2028 for 4 h. The internalization of QD_green_-C-2028 in cells was explored by CLSM. Scale bar 10 µm. (**b**) MFI values of the panel (**a**) were determined using ImageJ software and normalized to control (cells treated QD_green_-C-2028 without inhibitor). Data are expressed as the mean ± standard deviation of four independent experiments. * *p* < 0.05, ** *p* < 0.01, *** *p* < 0.001—statistically significant differences between the MFI of UAs and QDs in the cells incubated with inhibitors and control cells (no inhibitor) (Student’s *t*-test).

Figure 4 shows the changes in the internalization of QD_green_–C-2028 into cells treated with different endocytosis inhibitors. The internalization of QD_green_–C-2028 to H460, HCT116, and MRC-5 cells was highly energy-dependent. The penetration of QD_green_-C-2028 into H460 cells treated with Cytochalasin D was similar to the control (without inhibitor). In contrast, preincubation with Amiloride, Dynasore, Pitstop 2, and Filipin III significantly decreased the cellular uptake of QD_green_-C-2028 into cells. On the other hand, the internalization of QD_green_–C-2028 into HCT116 cells significantly decreased only in cells treated with Dynasore and Pitstop 2, which are inhibitors of CME. In the case of other inhibitors, the cellular uptake of QD_green_–C-2028 into HCT116 cells was similar to control cells (without inhibitors). Preincubation of MRC-5 cells with Cytochalasin D, Dynasore, Pitstop 2, and Filipin III, as opposed to Amiloride, led to significantly decreased cellular uptake of QD_green_–C-2028, but less than in cancer H460 cells.

### 3.3. Cell Cycle Analysis 

**Figure 5:**
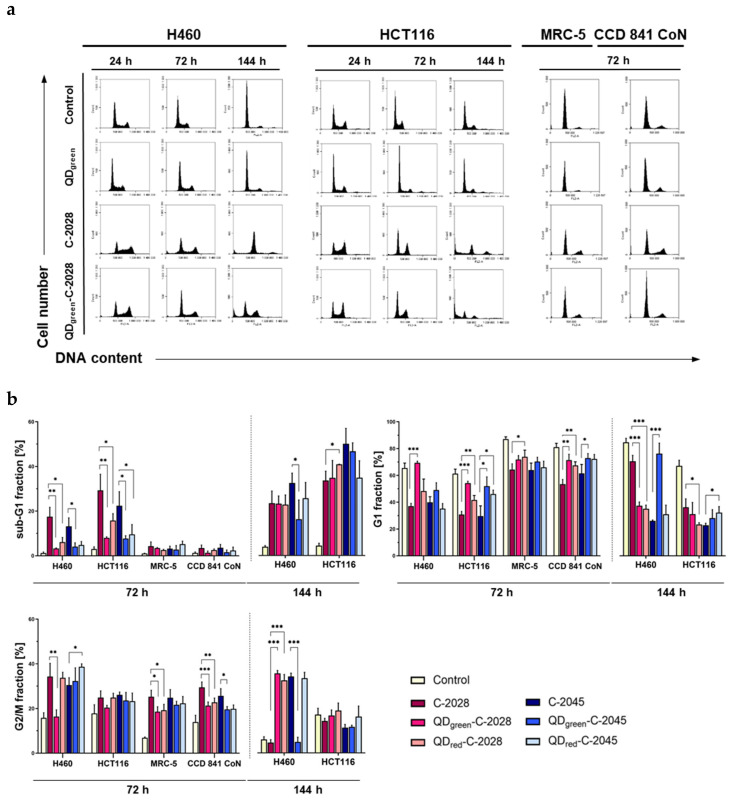
Cell cycle analysis of H460, HCT116, MRC-5, and CCD 841 CoN cells following treatment with QDs, UAs, and QD−UA hybrids for the time indicated. Cells were fixed in ethanol and stained with propidium iodide (PI), and their DNA content was measured by flow cytometry. (**a**) Representative plots of cells treated with selected QDs, UAs, and QD−UA hybrids. (**b**) Quantification of data shows the cell distributions in the sub-G1, G1, and G2/M phases of the cell cycle. Data represented the averages of three independent experiments. * *p* < 0.05, ** *p* < 0.01, *** *p* < 0.001 indicates statistically significant differences between fraction of cells incubated with UAs alone and QD−UA hybrids (Student’s *t*-test).

The impacts of UAs and QDs–UAs on the cell cycle progression of lung and colon cancer cells was measured by flow cytometry. Treatment of lung H460 cells with the C-2028 derivative alone and hybrids with QDs (green and red) resulted in a time-dependent increase in the population of hypodiploid cells (<2n content, sub-G1 fraction), representing the cells undergoing cell death (apoptosis). This population reached the same level (about 22%) following 144 h of incubation with QDs–C-2028 and with C-2028 alone (Figure 5a,b, Figure S3a and Table S1a). Exposure of H460 cells to QDs–C-2028 and C-2028 unbound led to a decrease in G1 population (about 33–35% and 67%, respectively, versus 80% in control cells). In addition, an accumulation of the cells in G2/M phase was detectable after 144 h of treatment with QDs–C-2028 (30–33% versus 5.6% in control cells).

In turn, the population of the G2/M phase gradually decreased following incubation with C-2028 alone and reached a similar level as in control cells (about 4.3%). Another bisacridine, the C-2045 derivative, also caused an increased number of cells in the sub-G1 phase (30% after 144 h), concomitantly with the reduction of cells in G1 phase (about 25% versus 80% in control cells) and an increase in the number of cells in G2/M phase (about 32% versus 6% in control; Figure 5b, Figure S3a, Table S1a). In turn, the number of cells in sub-G1 phase following treatment with QD–C-2045 hybrids were smaller, compared to C-2045 alone (15% for QD_green_–C-2045 and 24% for QD_red_–C-2045).

In HCT116 cells, the transient accumulation of cells in G2/M phase was observed after 24 h of treatment with both UA derivatives (C-2028 and C-2045), as well as with QDs-UAs (an increase from 26% in control to 40–53% in drug-treated cells). Following longer incubation times (up to 144 h), the number of cells in this phase decreased with a concomitant increase in the sub-G1 population (31%, 32%, 38%, 46%, 43%, and 32% following incubation with C-2028, QD_green_–C-2028, QD_red_–C-2028, C-2045, QD_green_–C-2045, and QD_red_–C-2045, respectively). Furthermore, the population of polyploid cells (with DNA more than 4n) gradually increased with incubation time (from 8% in control cells), to nearly 16–20% after 144 h of drug incubation (Figure 5, Figure S3a and Table S1b). However, the population of polyploid cells did not change in H460 cells following treatment with the studied drugs (Figure S3a).

For comparison, the hybrids of UAs with QDs did not significantly change the profiles in the histograms obtained by cell cycle distribution analysis of normal MRC-5 and CCD 841 CoN cells, compared to cancer H460 and HCT116 cells (Figure 5, Figure S3b and Table S1c,d). The changes in the cell cycle distribution of normal cells were visible only in the case of unbound bisacridines. Namely, in MRC-5 cells, both bisacridines caused the reduction of the number of cells in G1 phase (from 87% in control to 64%) with a simultaneous increase in the number of cells in G2/M phase (to 25% versus 7% in control). Similar effects were observed in CCD 841 CoN cells, where the G1 population decreased from 81% in control to 53% following treatment with C-2028 and 62% after incubation with C-2045. In turn, the G2/M population increased from 14% in control to 30% and 26% following treatment with C-2028 and C-2045, respectively. Additionally, the polyploid population also increased after incubation with C-2028 (from 2% in control to 13%). Importantly, QD_green_ and QD_red_ alone did not cause any changes in the cell cycle progression of tumor H460 and HCT116 cells, as well as normal MRC-5 and CCD 841 CoN cells (Figure 5, Figure S3a,b, and Table S1a–d).

### 3.4. Induction of Apoptosis 

**Figure 6:**
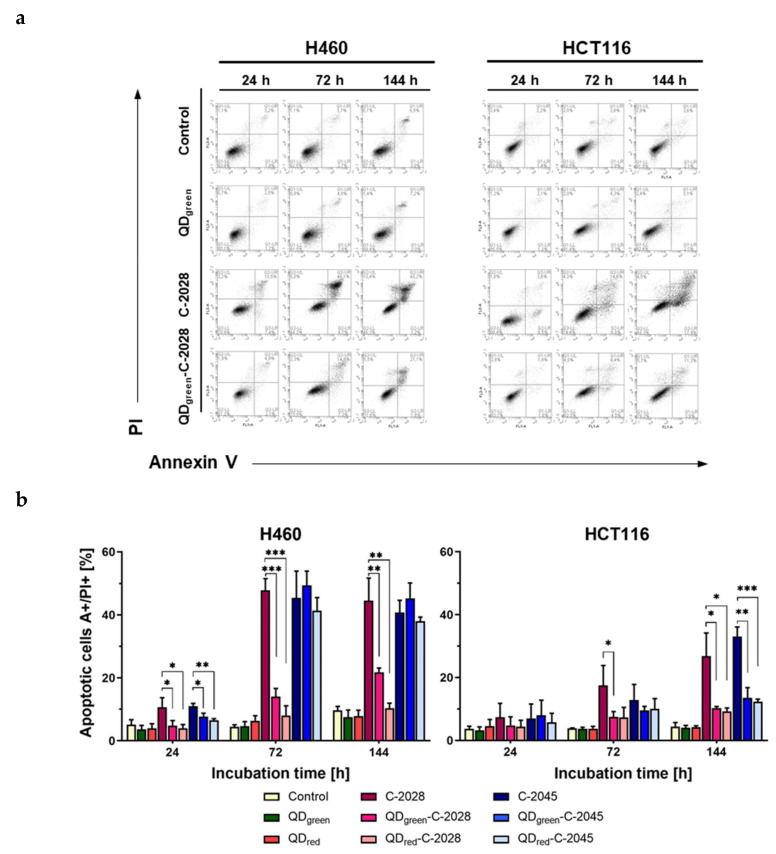
Flow cytometry analysis of phosphatidylserine externalization by Annexin V/propidium iodide (PI) assay in H460 and HCT116 cells. (**a**) Representative plots of cells treated with selected QDs, UAs, and QD−UA hybrids. (**b**) Quantification of data expressed as the percentage of late apoptotic cells (A+/PI+). Data represented the averages of three independent experiments. * *p* < 0.05, ** *p* < 0.01, *** *p* < 0.001 indicate statistically significant differences between the fraction of late apoptotic cells incubated with UAs alone and QD−UA hybrids (Student’s *t*-test). Bottom left quadrant represents live cells (Annexin V negative, PI negative); bottom right quadrant—early apoptotic cells (Annexin V positive, PI negative); top right quadrant—late apoptotic cells (Annexin V positive, PI positive); top left quadrant—primary necrotic cells (Annexin V negative, PI positive).

The presence of sub-G1 populations in cancer H460 and HCT116 cells prompted us to study apoptotic cell death in more detail. Flow cytometric analysis with Annexin V/PI (propidium iodide) dual staining was used to determine the cell death process induced by UAs alone and those hybrids with QDs in H460 and HCT116 cancer cells. As shown in Figure 6, Figure S4, and Table S2a,b (in the SM), the studied compounds (UAs) induced time-dependent apoptotic cell death in both cell lines, which reached a higher level in H460 than in HCT116 cells. The percentages of late apoptotic cells (A+/PI+) reached about 41–45% in H460 cells and about 27–33% in HCT116 cells treated with unbound UAs for 144 h. Linkage of UAs with QDs decreased the number of late apoptotic cells in both H460 and HCT116 cell lines, except for QD–C-2045 hybrids, which caused late apoptosis of H460 cells at the same level as the unbound C-2045 derivative. In contrast, cancer cells treated with QD_green_ and QD_red_ were Annexin V- and PI-negative (Annexin V-/PI-), indicating that they were viable and not undergoing apoptosis.

**Figure 7:**
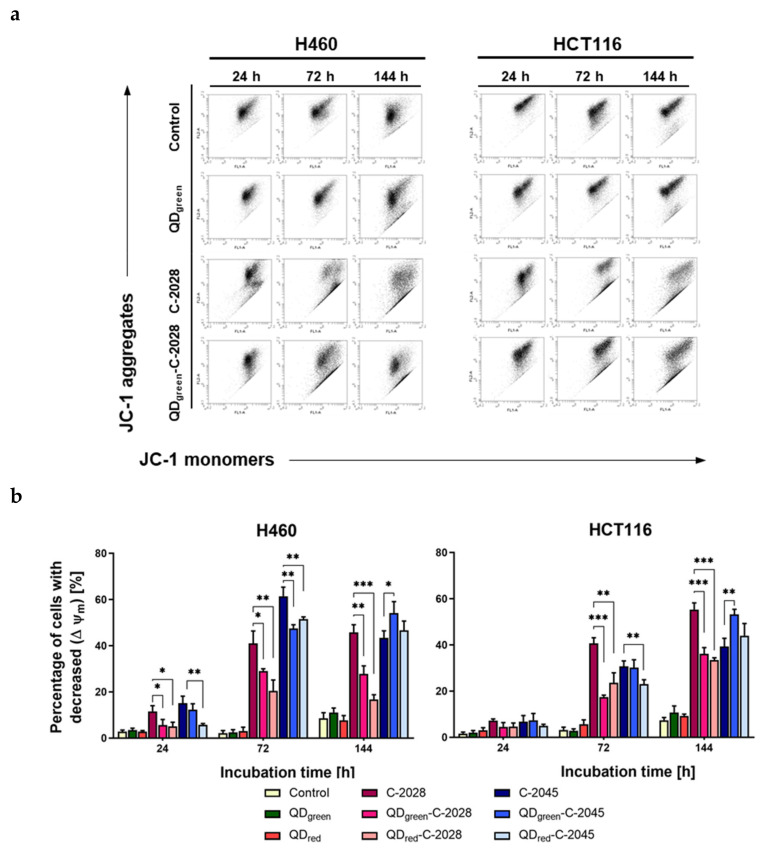
Analysis of the changes in mitochondrial membrane potential (ΔΨ_m_) in H460 and HCT116 cells following treatment with QDs, UAs, and QD−UA hybrids for 24, 72, and 144 h. ΔΨ_m_ was measured by flow cytometry using JC-1 dye. (**a**) Representative plots of cells treated with selected QDs, UAs, and QD−UA hybrids. (**b**) Quantification of data expressed as the percentage of cells with decreased ΔΨ_m_. Data represented the averages of three independent experiments. * *p* < 0.05, ** *p* < 0.01, *** *p* < 0.001 indicate statistically significant differences between the percentage of cells with decreased ΔΨ_m_ incubated with UAs alone and QD−UA hybrids (Student’s *t*-test).

The induction of apoptosis by UAs and their hybrids with QDs was also confirmed by measuring the disruption of the mitochondrial membrane potential (MMP). Changes of MMP in the H460 and HCT116 cells following treatment with QDs, UAs, and QD−UA hybrids were determined by measuring the changes of the emitted fluorescence variation form of JC-1 dye using flow cytometry. JC-1 dye selectively aggregates in normally polarized mitochondria and emits red fluorescence. In apoptotic depolarized cells, where the MMP is reduced, the JC-1 dye assumes a monomeric form in the cytosol and emits green fluorescence. As shown in Figure 7, Figure S5, and Table S3a,b (in the SM), the UAs induced time-dependent loss of mitochondrial membrane potential (ΔΨ_m_) in both H460 and HCT116 cell lines through decreasing red and increasing green fluorescence of JC-1. No significant changes in the emission of fluorescence of JC-1 dye in both H460 and HCT116 cells treated with free QDs, compared to control cells (untreated cells), were observed. UAs non-covalently attached to QDs (QD−UA hybrids) significantly decreased the amount of monomer form of JC-1 dye (green fluorescence) in H460 and HCT116 cells, compared to unbound UAs, except for cells treated with QDs-C-2045 for 144 h of incubation; in this case, the number of cells with depolarized mitochondria reached the same level as that following treatment with C-2045 alone.

### 3.5. Induction of Cellular Senescence 

**Figure 8:**
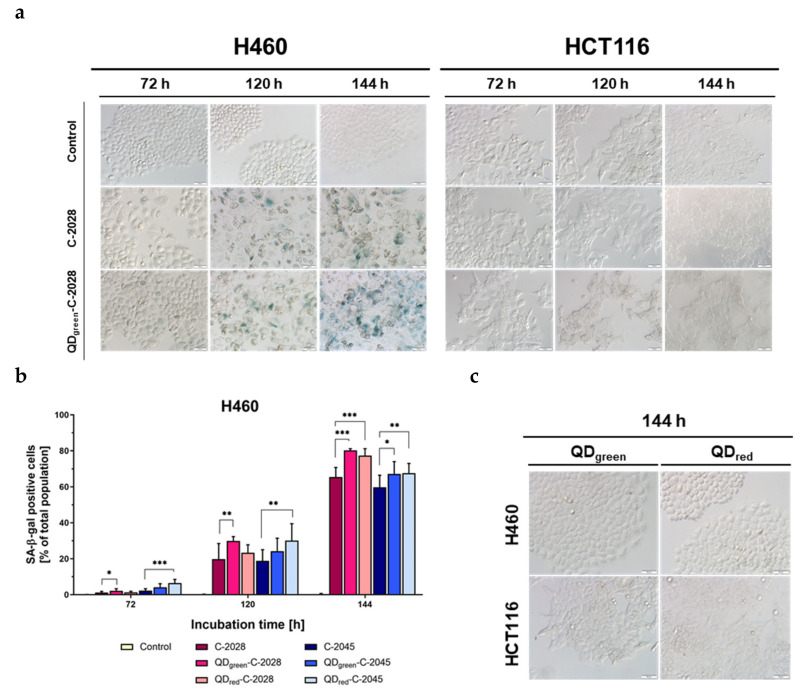
Cellular senescence of H460 and HCT116 cancer cells following treatment with QDs, UAs, and QD−UA hybrids for the time indicated. Senescence-associated β-galactosidase activities were assessed by X-gal staining using a light microscope. (**a**) Representative images of cells treated with selected UAs and QD−UA hybrids. (**b**) Quantification of data expressed as the percentage of SA-β-gal positive cells in H460 cells. (**c**) Representative images of cells treated with QDs in H460 and HCT116 cells. Data represented the averages of three independent experiments. * *p* < 0.05, ** *p* < 0.01, *** *p* < 0.001 indicate statistically significant differences between the percentage of SA-β-gal positive cells incubated with UAs alone and QD−UA hybrids (Student’s *t*-test). The scale bar 50 µm.

As shown above, both studied bisacridines and their hybrids with QDs induced apoptosis in cancer cells; more efficiently in lung H460 cells than in HCT116 cells. Moreover, the process of cell death affected only part of the cancer cell population. Therefore, in the next step of our studies, we examined whether our compounds induced cellular senescence. The expression of SA-β-gal (SA-β-galactosidase) and morphological features, especially enlargement with abundant granulation and flattened cell shape, were used as markers of senescence. The characteristic blue color resulting from the metabolic activity of the SA-β-gal substrate was observed in H460 cells from 72 h of incubation with UAs (Figure 8 and Figure S6 in the SM). The enhanced expression of senescence-associated SA-β-galactosidase was also observed following treatment of H460 cells with QD−UA hybrids. This effect was increased following prolonged incubation times (up to 144 h) and the number of senescence-positive cells was higher with QDs–C-2028 hybrids than for C-2028 alone. Interestingly, both UAs and QDs–UAs did not induce senescence in HCT116 cells. Furthermore, QD_green_ and QD_red_ (QDs) did not induce senescence in both H460 and HCT116 cancer cells after 144 h of incubation (Figure 8c). 

## 4. Discussion

We found that the application of QDs as a delivery platform for unsymmetrical bisacridines to cells affected the cellular uptake and biological response in cancer and normal cells. The results of the presented research indicate that UAs non-covalently attached to the QDs, enhancing the amount of drug in the cells (compared to UAs alone), especially in the case of QDs–C-2028, in human lung H460 cells (Figure 3 and Figure S2a–d). Furthermore, in these cells, more QD−UA hybrids were accumulated following prolonged incubation time, in contrast to human colon HCT116 and normal MRC-5 cells. In these cells, the highest fluorescence intensity of QD−UA hybrids was observed following 24 h of incubation and, after that time, the fluorescence intensity of the hybrids started to decrease. The observed enhanced cellular uptake and the phenomenon of more effective accumulation of QDs–UAs in H460 cells than in HCT116 cells were consistent with our previous results [22]. We showed that the linkage of UAs with QDs significantly improved the cytotoxic activity of these drugs, especially in H460 cells, and led to a protective effect on normal MRC-5 and CCD 841 CoN cells. Moreover, UAs and QD−UA hybrids, after cellular internalization, were localized in lysosomes (where the pH value promoted the greater release of UAs from the QD−UA hybrids) more effectively in cancer cells than in normal cells, due to the differences in pH between these cells [25]. The increased cellular uptake of QDs–UAs, compared to UAs alone, in the H460 cells investigated here, further confirmed their higher cytotoxicity against these cells. 

The internalization mechanism study (Figure 4) indicated that QD_green_–C-2028 hybrids in H460 cells (and slightly weaker in MRC-5 cells) were taken up by three endocytosis pathways: CME, CavME, and MP. Similar mechanisms of endocytosis have been observed by Nagai N. et al. [26], who showed that ketoprofen nanoparticles (KET-NPs) penetrate cells by three endocytosis pathways (CavME, CME, and MP). In another study, it has been shown that DPA-QDs (DPA, D-penicillamine) were mainly internalized through CME (mostly by the dynamin-dependent pathway) and MP [27]. In turn, in the case of HCT116 cells, we showed that QD_green_–C-2028 hybrids were taken up only by one endocytosis pathway, CME. Furthermore, Dynasore (an inhibitor of CME) significantly reduced the uptake of QD_green_–C-2028 hybrids, most efficiently in the case of H460 cells. In receptor-mediated endocytosis, also called CME (clathrin-mediated endocytosis), two main factors affect the endocytosis process. The first one is the available receptors on the cell membrane (e.g., folic acid and transferrin receptors), which are often overexpressed in cancer cells. The second one is non-specific absorbed proteins on the surface of NPs (e.g., α- and β-globulin proteins) [28]. These factors can determine whether and how NPs enter into the cells by CME, as well as affecting the uptake of NPs into cancer cells through multiple receptors on the cell membrane. Moreover, the mechanism of the internalization of NPs into cells depends on several variables, including the cell type, particle size, material composition, shape, and surface properties [29]. The multiple pathway internalization of QD−UA hybrids (especially QDs–C-2028) can provide an explanation of their higher cellular uptake and cytotoxicity in H460 cells, compared to UAs alone.

It has been well-established that cell cycle arrest and apoptosis are prime therapeutic targets for cancer treatment [30,31]. Various chemotherapeutics have been shown to induce apoptosis and G2/M phase arrest, including monomeric acridine derivatives such as imidazoacridinones and 1-nitroacridines [32,33,34,35]. We have also shown that Doxorubicin covalently bound to magnetic nanoparticles (Dox-Nps) induces G2/M phase arrest in the human colon HT29 cells, followed by apoptosis and necrosis [36]. In this paper, we demonstrated that unsymmetrical bisacridines and their hybrids with quantum dots induce apoptosis in human lung H460 and colon HCT116 cells, as revealed by Annexin V/PI staining and changes in mitochondrial membrane potential (ΔΨ_m_) (Figure 6, Figure 7, Figures S4 and S5). However, a larger population of apoptotic cells (A+/PI+, green fluorescence of JC-1) was observed following free C-2028 treatment, in comparison to its hybrids. In the case of the C-2045 derivative, apoptosis was induced on the same level as by QDs–C-2045. Furthermore, the induction of apoptosis by both UAs and their hybrids was much more potent in H460 cells than in HCT116 cells. These data were consistent with the cell cycle analysis results, which indicated an increasing sub-G1 population in both human cancer cell lines following UAs and their hybrids treatment, which was greater in lung H460 cells. Importantly, even following long incubation times (up to 72 h) with unbound UAs and their hybrids with QDs, the sub-G1 population remained at a very low level in normal cells (Figure 5 and Figure S3a,b). This observation may indicate that unsymmetrical bisacridines, as well as their quantum dot hybrids, do not significantly affect the behavior of normal cells. This is consistent with our previous results, which clearly showed that noncovalently attached UAs to quaternary quantum dots improved cytotoxicity in H460 cancer cells, while decreasing cytotoxicity in normal cells [22]. It is worth emphasizing that free QDs did not induce significant changes in the cell cycle progression and did not induce apoptosis in tumor cells, as well as in normal cells. This is a serious advantage of these nanoparticles, in terms of their potential use as a platform for drug delivery to cancer cells.

Further, we examined whether UAs and QDs–UAs treated cancer cells that did not die by apoptosis underwent cellular senescence. The linkage of UAs with QDs significantly increased the number of H460 senescent cells, compared to cells treated with UAs alone (Figure 8a,b and Figure S5). Moreover, H460 cells were more prone to undergoing senescence following treatment with QDs-C-2028 hybrids. Surprisingly, HCT116 cells did not undergo senescence following either UAs hybrids or by free UAs treatment. Importantly, QDs alone did not induce senescence in either H460 or HCT116 cancer cells (Figure 8c). To the best of our knowledge, no previous reports have investigated the influence of anticancer drugs conjugated with QDs on the induction of cellular senescence. Further, only a few studies have shown the influence of drugs (e.g., doxorubicin; DOX) conjugated with other NPs on the induction of senescence [37]. Lazaro-Carrillo A. et al. showed that iron oxide magnetic NPs (MF66) alone did not induce senescence in MDA-MB-231 cancer cells. Besides, they found that cells treated with MF66–DOX conjugates, compared to MF66 alone, induced senescence, apoptosis, and mitotic catastrophe. Therefore, we emphasize that this is a pioneer study examining the effects of QD–UA hybrids on the senescence process in tumor cells. The increase of senescence in H460 cancer cells may be another mechanism contributing to the anticancer effect of QD-UA hybrids. Different morphological changes in H460 cancer cells (e.g., apoptotic cells or enlarged and flattened cell shapes characteristic of senescent cells) were confirmed by time-lapse recording. The full-length movies are available in the Supplementary Materials (Videos S1–S3).

## 5. Conclusions

In this study, we presented a nanoparticle-based drug delivery system using quantum dots (QDs) conjugated with unsymmetrical bisacridines (UAs), which have high antitumor activity. CLSM analysis showed the increased cellular uptake of QD–UA hybrids, compared to UAs alone, in human lung H460 cells, which may have resulted from the co-operation of three endocytosis pathways: CME, CavME, and MP. In turn, in the case of HCT116 cells, QDs–UAs were taken up only by one endocytosis pathway (CME) and the accumulation of the studied compounds and their hybrids was significantly weaker. These results confirmed our previous research findings on the higher cytotoxic activity of QDs–UAs, compared to UAs alone, in H460 cells [22]. Flow cytometry analysis revealed that both QDs–UAs and free UAs induced time-dependent apoptosis in both studied cell lines, which was more efficient in H460 cells. However, the linkage of UAs with QDs decreased the number of late apoptotic cells in both cell lines, except for the C-2045 derivative, the hybrids of which induced cell death in H460 cells at the same level as the unbound compound. Importantly, both hybrids and free UAs did not induce apoptosis (sub-G1 population) in normal MRC-5 and CCD-841 CoN cells, indicating their protective effect against these cells. Moreover, unsymmetrical bisacridines and their hybrids induced cellular senescence in H460 cells; this process was much more effective in the case of QD–UA hybrids. Interestingly, HCT116 cells did not undergo cellular senescence following QDs–UAs or UAs treatment. It is worth pointing out that free QDs did not affect the processes of apoptosis and senescence in tumor cells, as well as in normal cells. In summary, the described results clearly demonstrated that QDs may serve as a good platform for drug delivery to cancer cells.

## Data Availability

Not applicable.

## Supplementary Materials

The following are available online at https://www.mdpi.com/2079-4991/11/2/462/s1, Figure S1. Fluorescence spectra of QD_green_ (a) and QD_red_ (b) nanocrystals and their hybrids with C-2028 and C-2045. Insets: fluorescence spectra of C-2028 and C-2045. Experimental conditions: C_UAs_ = 300 µM; C_QDs-UAs_ = 1 mg∙mL^−1^; PMT voltage: 700 V; PMT integration time: 20 ms; excitation wave: 425 nm, Figure S2. Cellular uptake of QDs, UAs and QD−UA hybrids to cancer ((a) H460 and (b) HCT116) and normal ((c) MRC-5 and (d) CCD 841 CoN) cells for the time indicated, Figure S3. cell cycle analysis of H460, HCT116, MRC-5, and CCD 841 CoN cells following treatment with QDs, UAs, and QD−UA hybrids for the time indicated, Figure S4: Induction of apoptosis in H460 and HCT116 cells following treatment with QDs, UAs, and QD−UA hybrids for the time indicated, Figure S5: Analysis of the changes in mitochondrial membrane potential (ΔΨ_m_) decreasing in H460 and HCT116 cells following treatment with QDs, UAs, and QD−UA hybrids, Figure S6: Cellular senescence of H460 and HCT116 cancer cells following treatment with UAs and QD−UA hybrids for the time indicated. Senescence-associated β-galactosidase activities were assessed by X-gal staining using light microscope. Data represented the images of three independent experiments. The scale bar 50 µm. Table S1. Analysis of cell cycle by flow cytometry in (a) H460, (b) HCT116, (c) MRC-5, and (d) CCD 841 CoN cells. Data shows percentage of cells treated with QDs, UAs, and QD−UA hybrids at IC_80_ value for the time indicated in the sub-G1, G1, S, G2/M, and Poli (polyploid cells) phases of the cell cycle. Data represented the averages of three independent experiments. Table S2. Flow cytometry analysis of phosphatidylserine externalization by Annexin V/propidium iodide (PI) assay in (a) H460 and (b) HCT116 cells. Percentage of cells treated with QDs, UAs, and QD−UA hybrids at IC_80_ value. Data represented the averages of three independent experiments. A-/PI- (Annexin V negative, PI negative) represents live cells; A+/PI- (Annexin V positive, PI negative)—early apoptotic cells; A+/PI+ (Annexin V positive, PI positive)—late apoptotic cells; A-/PI+ (Annexin V negative, PI positive)—primary necrotic cells. Table S3. cytometric analysis of changes in mitochondrial transmembrane potential (ΔΨ_m_) in (a) H460 and (b) HCT116 cells. Cell were treated with QDs, UAs, and QD−UA hybrids at IC_80_ value for the time indicated. Data shows percentage of cells with decreased mitochondrial transmembrane potential (ΔΨ_m_) (green fluorescence). Data represented the averages of three independent experiments. Supplementary Movies S1–S3: Time-lapse imaging of H460 cells incubated with (1) control, (2) QD_red_, (3) QD_red_-C-2028.
