# Supplementary material for: Quantum Dots as a Good Carriers of Unsymmetrical Bisacridines for Modulating Cellular Uptake and the Biological Response in Lung and Colon Cancer Cells
Source: Nanomaterials (Basel). 2021 Feb 11;11(2):462. doi: 10.3390/nano11020462 (PMC7917955; doi:10.3390/nano11020462)
Supplement: Supplementary file 1 [file nanomaterials-11-00462-s001.zip › Supplementary Materials/nanomaterials-1097572-supplementary.pdf]

nanomaterials

Supplementary Materials

Quantum Dots as a Good Carriers of Unsymmetrical Bisacridines for Modulating Cellular Uptake and the Biological Response in Lung and Colon Cancer Cells

Joanna Pilch [1,*], Patrycja Kowalik [2,3], Piotr Bujak [3], Anna M. Nowicka [2] and Ewa Augustin [1,*]

[1] Faculty of Chemistry, Gdańsk University of Technology, Narutowicza Str. 11/12, 80-233 Gdańsk, Poland
[2] Faculty of Chemistry, University of Warsaw, Pasteura Str. 1, 02-093 Warsaw, Poland; pkowalik@ch.pw.edu.pl (P.K.); anowicka@chem.uw.edu.pl (A.M.N.)
[3] Faculty of Chemistry, Warsaw University of Technology, Noakowskiego Str. 3, 00-664 Warsaw, Poland; piotrbujakchem@poczta.onet.pl
* Correspondence: joapilch@pg.edu.pl (J.P.); ewa.augustin@pg.edu.pl (E.A.); Tel.: +48-58-347-12-97 (J.P.); +48-58-347-14-68 (E.A.)

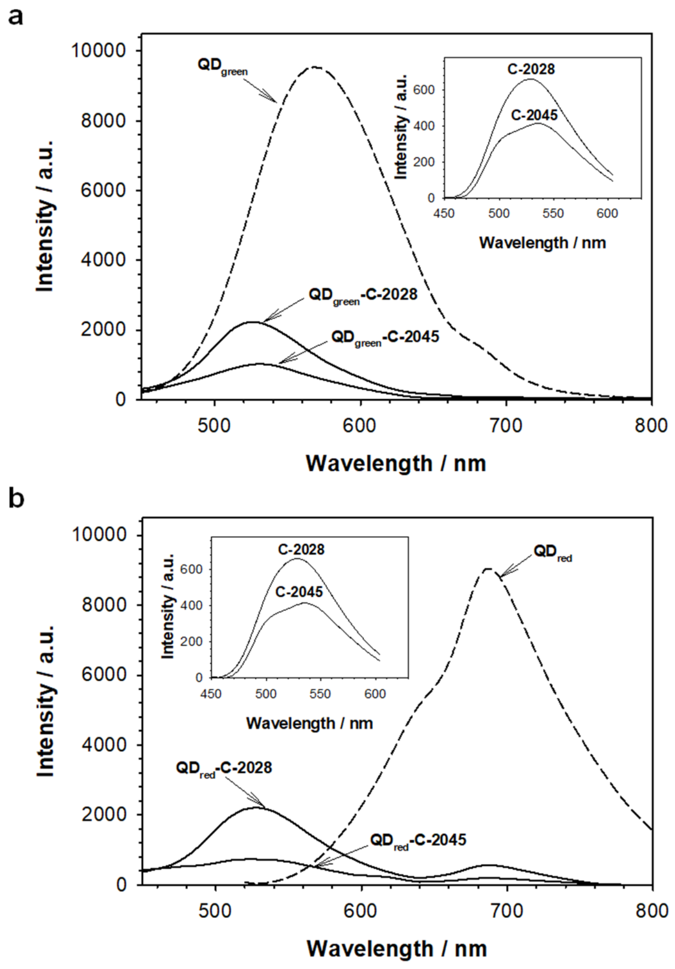

Figure S1. Fluorescence spectra of QD_{green} (**a**) and QD_{red} (**b**) nanocrystals and their conjugates with C-2028 and C-2045. Insets: fluorescence spectra of C-2028 and C-2045. Experimental conditions: C_{UAs} = 300 μM; $C_{QDs\text{-}UAs}$ = 1 mg·mL^{-1}; PMT voltage: 700 V; PMT integration time: 20 ms; excitation wave: 425 nm.

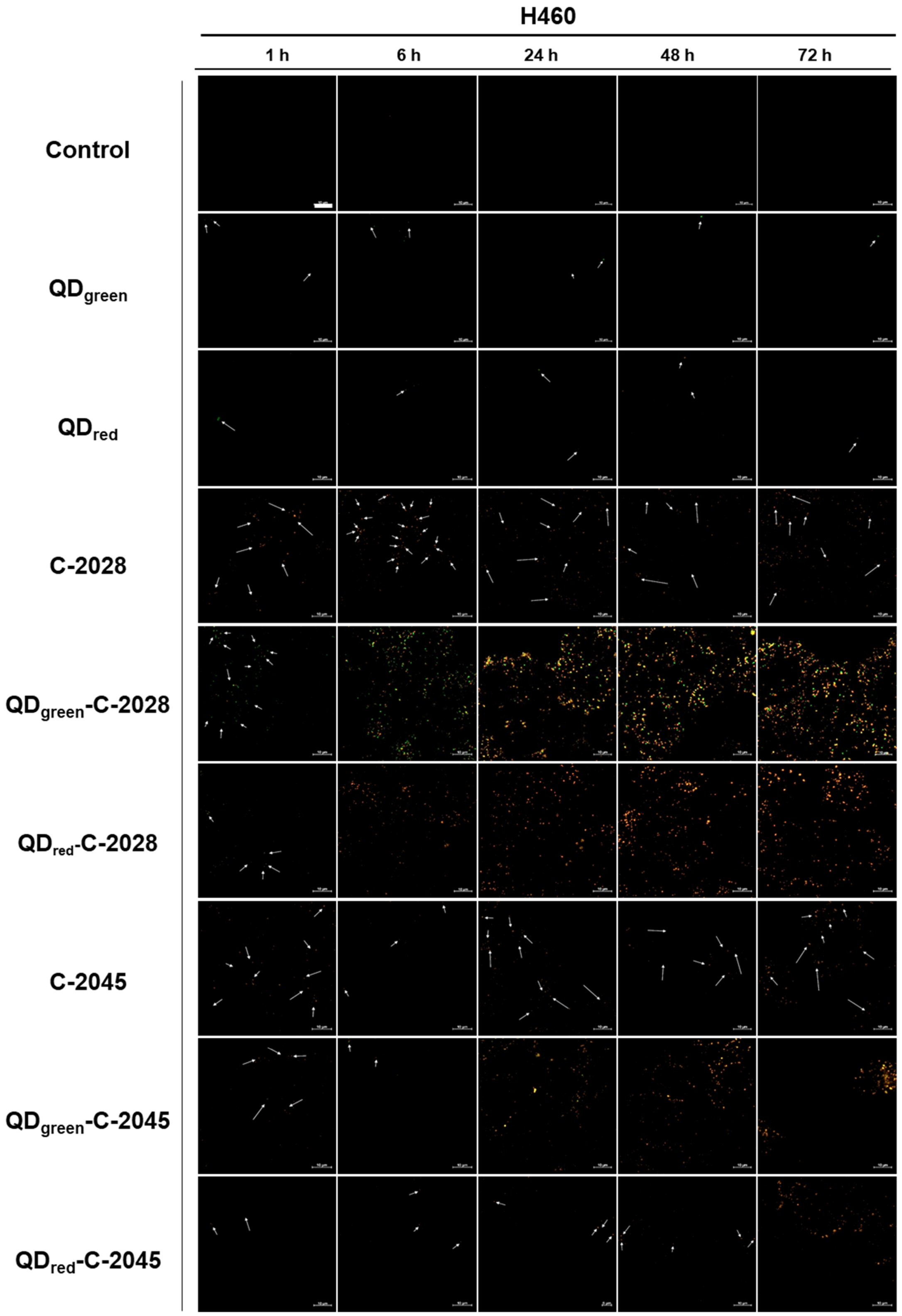
H460
1 h
6 h
24 h
48 h
72 h
Control
QDgreen
QDred
C-2028
QDgreen-C-2028
QDred-C-2028
C-2045
QDgreen-C-2045
QDred-C-2045

(**a**)

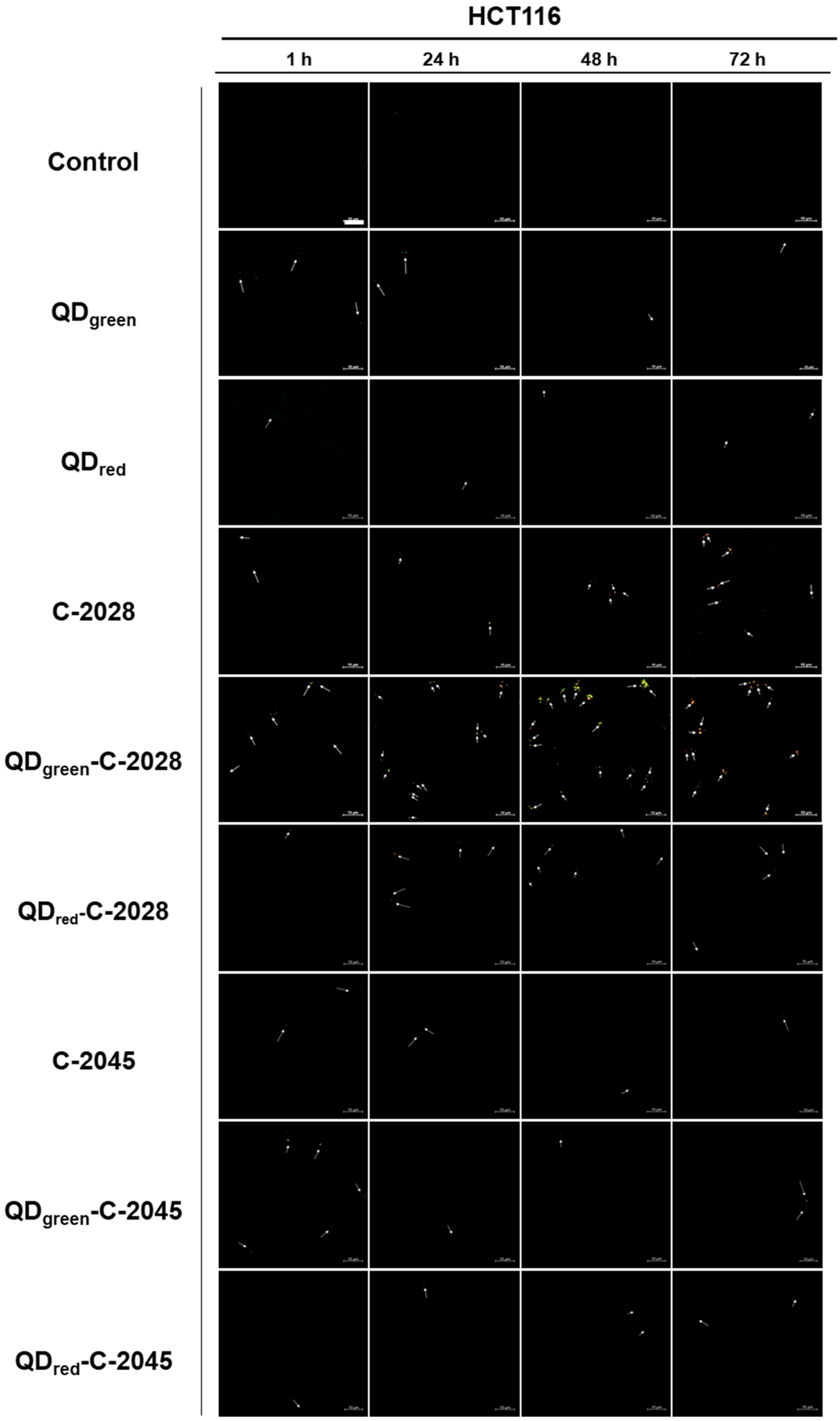
HCT116
1 h
24 h
48 h
72 h
Control
QD_{green}
QD_{red}
C-2028
QD_{green}-C-2028
QD_{red}-C-2028
C-2045
QD_{green}-C-2045
QD_{red}-C-2045

(**b**)

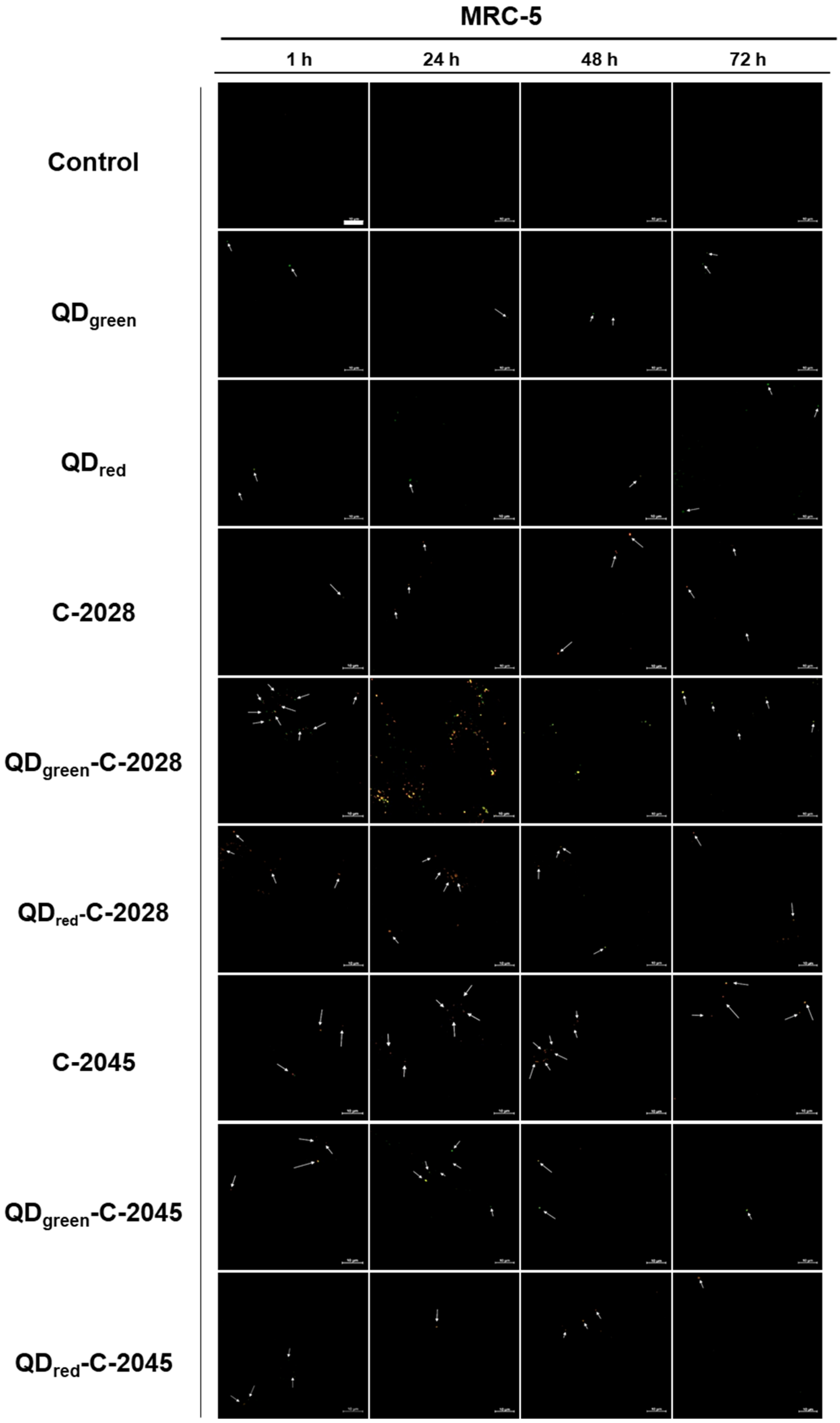
MRC-5
1 h
24 h
48 h
72 h
Control
QDgreen
QDred
C-2028
QDgreen-C-2028
QDred-C-2028
C-2045
QDgreen-C-2045
QDred-C-2045

(c)

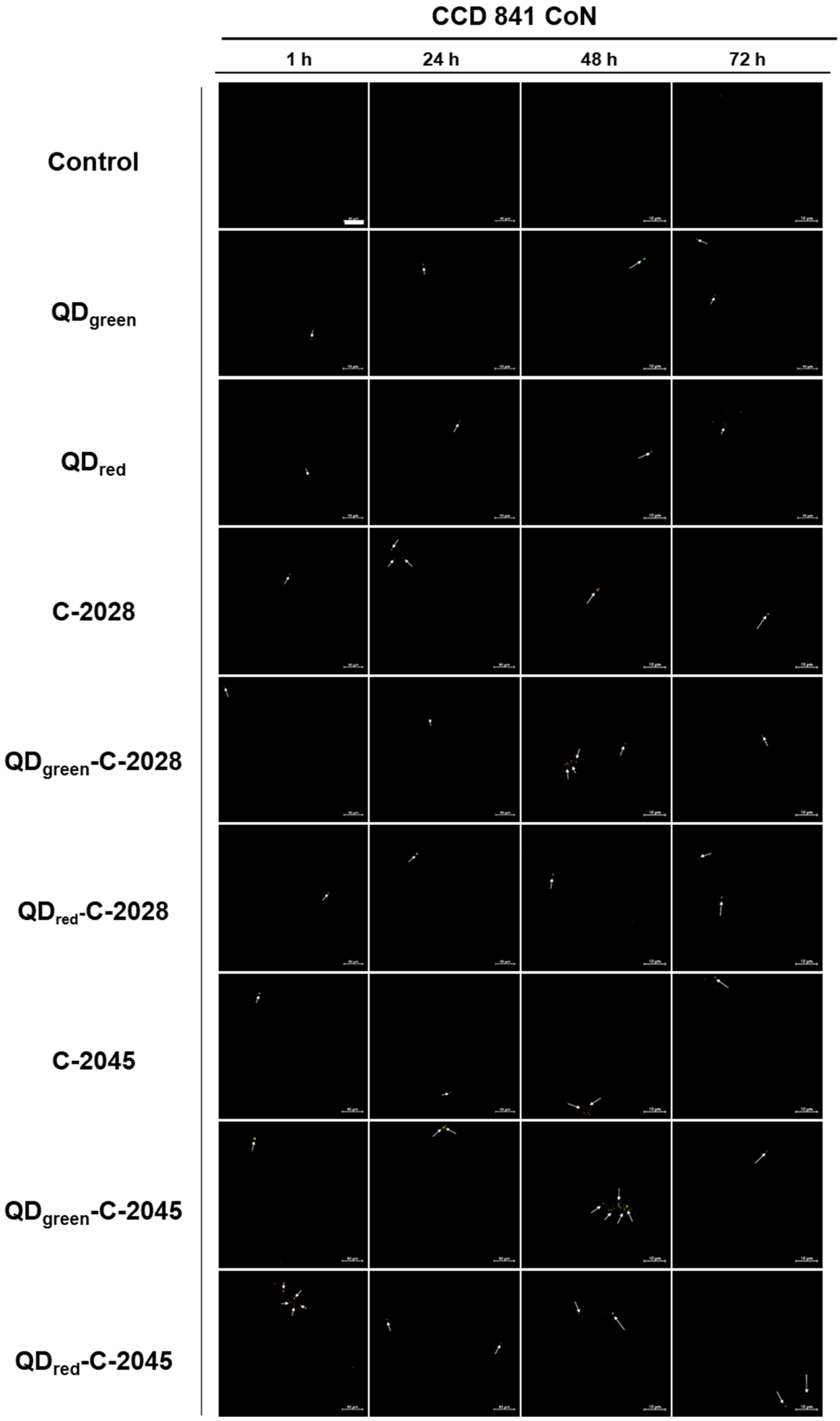

(**d**)

Figure S2 (**a**) Cellular uptake of QDs, UAs and QDs-UAs hybrids to cancer H460 cells for the time indicated and analyzed by CLSM. Scale bar 10 μm. Data represented the images of three independent experiments. (**b**) Cellular uptake of QDs, UAs and QDs-UAs conjugates to cancer HCT116 cells for the time indicated and analyzed by CLSM. Scale bar 10 μm. Data represented the images of three independent experiments. (**c**) Cellular uptake of QDs, UAs and QDs-UAs hybrids to normal MRC-5 cells for the time indicated and analyzed by CLSM. Scale bar 10 μm. Data represented the images of three independent

experiments. (**d**) Cellular uptake of QDs, UAs and QDs-UAs hybrids to normal CCD 841 CoN cells for the time indicated and analyzed by CLSM. Scale bar 10 μm. Data represented the images of three independent experiments.

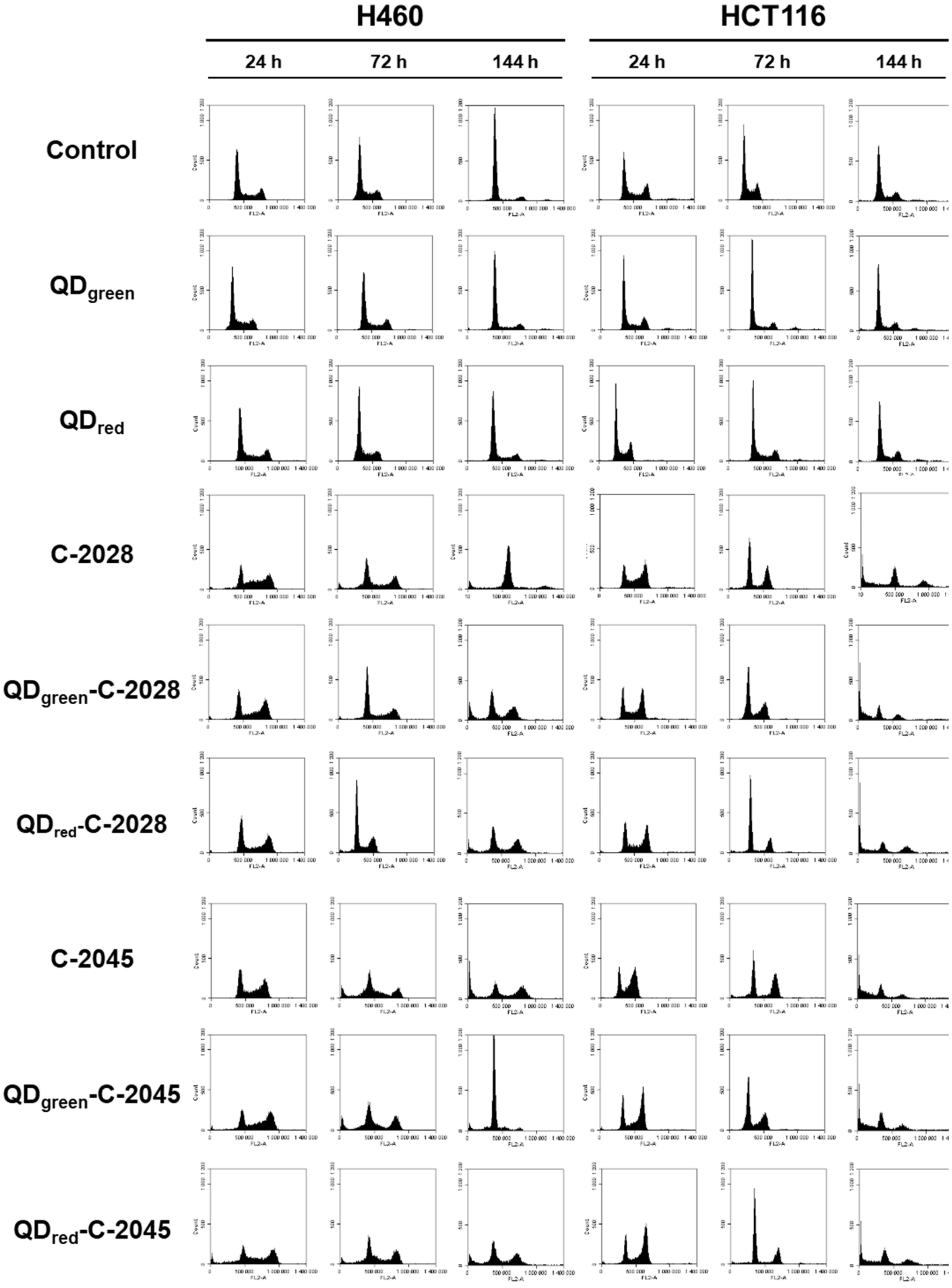

(**a**)

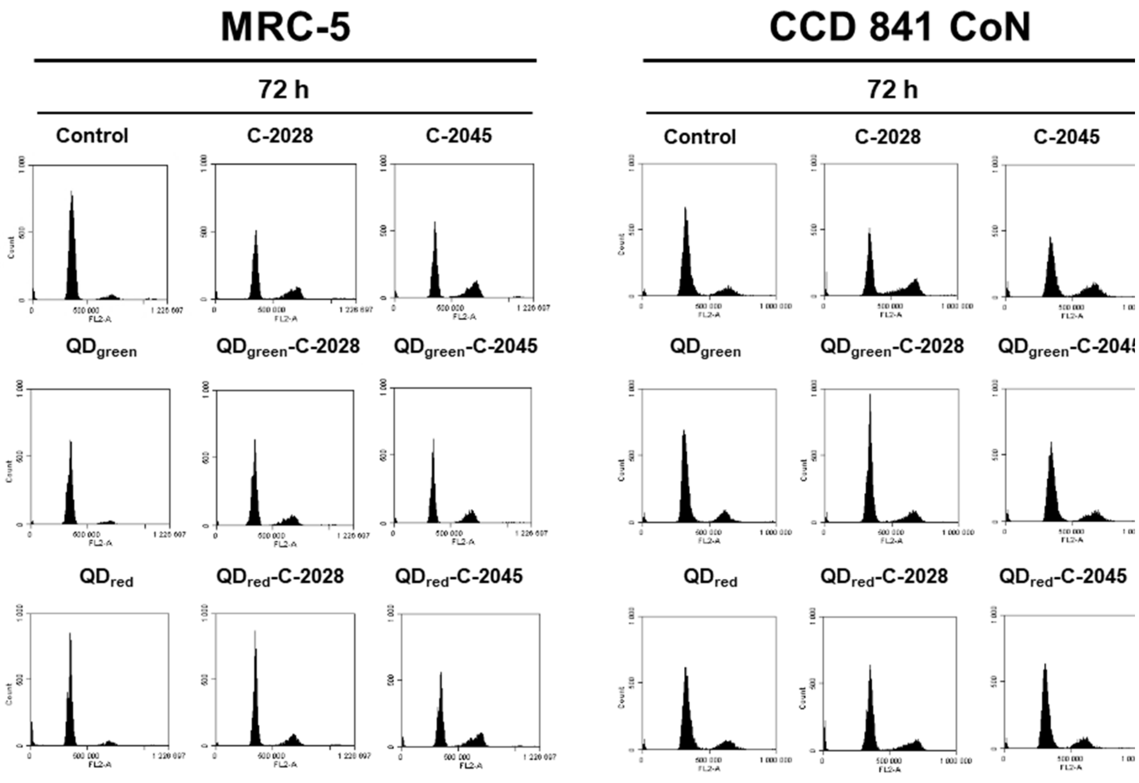

(**b**)

Figure S3 (**a**) Cell cycle analysis. Representative histograms of cancer H460 and HCT116 cells following treatment with QDs, UAs and QDs-UAs hybrids for the time indicated. Cells were fixed in ethanol and stained with PI, and their DNA content was measured by flow cytometry. Data represented the histograms of three independent experiments. (**b**) Cell cycle analysis. Representative histograms of normal MRC-5 and CCD 841 CoN cells following treatment with QDs, UAs and QDs-UAs hybrids for 72 h of incubation. Cells were fixed in ethanol and stained with PI, and their DNA content was measured by flow cytometry. Data represented the histograms of three independent experiments.

Table S1 a. Analysis of cell cycle by flow cytometry in H460 cells. Data shows percentage of cells treated with QDs, UAs and QDs-UAs hybrids at IC_{80} value for the time indicated in the sub-G1, G1, S, G2/M, and Poli (polyploid cells) phases of the cell cycle. Data represented the averages of three independent experiments.

Compound	[h]	Phases of the cell cycle									
		**sub-G1**	**SD**	**G1**	**±**	**S**	**±**	**G2/M**	**±**	**Poli**	**±**
**Control**	**24**	1.33	0.93	57.47	0.90	16.5	1.9	21.1	1.1	1.8	1.2
	**72**	1.23	0.47	65.5	3.7	15.63	0.75	15.8	2.3	1.8	1.2
	**144**	3.67	0.46	79.9	2.8	4.37	0.65	5.6	1.1	6.5	1.2
**QD_{green}**	**24**	0.80	0.17	60.33	0.21	16.2	3.1	20.0	3.0	2.67	0.72
	**72**	1.233	0.058	68.8	2.5	11.53	0.29	15.10	0.61	3.4	2.6
	**144**	2.87	0.76	76.1	8.6	6.0	3.6	10.1	4.6	4.9	3.5
**QD_{red}**	**24**	0.80	0.26	59.2	3.8	15.60	0.79	21.4	1.8	2.9	1.2
	**72**	1.233	0.058	69.57	0.35	11.97	1.70	14.6	1.7	2.6	1.3
	**144**	2.83	0.96	76.7	7.2	5.4	3.2	8.7	3.8	5.6	1.0

**C-2028**	**24**	3.7	1.6	34.3	5.3	30.33	0.87	29.5	5.1	2.1	2.7
	**72**	17.5	4.2	37.0	2.1	13.6	0.6	34.3	5.9	2.9	2.3
	**144**	21.7	5.0	66.6	4.1	3.5	1.6	4.3	1.2	4.88	2.11
**QD$_{green}$-C-2028**	**24**	2.37	0.64	41.9	4.4	19.03	0.60	36.4	3.4	0.30	0.35
	**72**	3.27	0.25	69.3	1.3	8.83	0.74	16.4	3.0	2.2	1.4
	**144**	21.5	3.2	35.3	2.6	7.60	0.85	33.0	1.2	2.6	1.4
**QD$_{red}$-C-2028**	**24**	2.17	0.55	35.4	2.7	26.4	3.9	35.3	1.5	1.8	2.9
	**72**	6.1	2.1	48.3	9.0	14.4	1.6	33.7	2.5	2.3	2.0
	**144**	21.1	4.0	33.2	2.5	9.97	0.81	30.1	2.4	5.6	1.3
**C-2045**	**24**	5.90	0.61	39.5	4.9	23.6	2.8	30.6	3.2	0.37	0.46
	**72**	13.2	3.8	39.9	4.2	17.6	1.1	30.5	3.3	2.70	0.42
	**144**	30.1	4.1	24.67	0.61	8.4	1.3	31.7	1.4	5.2	1.1
**QD$_{green}$-C-2045**	**24**	2.90	0.40	36.6	6.7	20.1	1.8	39.7	4.2	0.67	0.60
	**72**	4.1	1.7	49.1	5.4	14.7	3.3	32.3	5.9	2.1	1.2
	**144**	15.1	7.9	71.9	7.4	7.3	1.4	4.6	1.9	1.03	0.21
**QD$_{red}$-C-2045**	**24**	4.8	1.6	32.8	2.3	26.2	1.4	35.1	1.6	1.10	0.35
	**72**	9.7	1.3	35.2	3.8	13.3	1.8	38.7	1.3	4.55	0.21
	**144**	23.8	6.5	29.3	6.4	9.8	1.9	31.0	2.4	6.15	0.64

Table S1 b. Analysis of cell cycle by flow cytometry in HCT116 cells. Data shows percentage of cells treated with QDs, UAs and QDs-UAs hybrids at IC$_{80}$ value for the time indicated in the sub-G1, G1, S, G2/M, and Poli (polyploid cells) phases of the cell cycle. Data represented the averages of three independent experiments.

**Compound**		**Phases of the cell cycle**									
	**[h]**	**sub-G1**	**SD**	**G1**	**SD**	**S**	**SD**	**G2/M**	**SD**	**Poli**	**SD**
**Control**	**24**	2.00	0.10	46.4	5.0	17.5	1.3	26.2	1.3	10.3	1.6
	**72**	3.0	1.0	61.3	3.4	11.9	3.0	17.8	3.9	5.9	3.2
	**144**	4.1	1.0	63.3	4.0	9.1	2.0	15.9	2.6	7.6	2.3
**QD$_{green}$**	**24**	2.30	0.71	48.9	7.5	13.9	2.1	25.6	4.7	11.70	0.71
	**72**	2.33	0.67	56.0	3.1	13.23	0.65	20.7	1.1	6.20	0.71
	**144**	6.9	1.5	58.1	3.8	8.4	1.7	17.1	1.7	9.6	4.1
**QD$_{red}$**	**24**	1.40	0.36	53.5	5.3	15.1	1.3	26.0	5.5	4.8	2.6
	**72**	3.5	1.3	56.4	1.4	12.933	0.058	20.1	1.8	7.07	0.84
	**144**	3.67	0.49	63.4	4.7	9.5	1.0	17.33	0.75	6.1	3.8
**C-2028**	**24**	3.07	0.57	33.0	2.5	21.4	2.6	41.53	0.21	1.0	1.4
	**72**	29.3	7.2	30.8	2.3	6.83	0.60	24.9	3.0	8.2	4.3
	**144**	31.1	3.9	34.2	5.9	4.23	0.28	14.3	2.1	16.3	4.5
**QD$_{green}$-C-2028**	**24**	2.03	0.75	37.4	1.9	17.1	2.9	39.3	3.5	5.5	2.4
	**72**	8.07	0.46	54.2	1.5	5.80	0.87	20.4	1.0	10.2	1.5
	**144**	32.2	7.2	29.4	8.2	4.1	1.6	15.5	2.3	18.7	7.3
**QD$_{red}$-C-2028**	**24**	1.53	0.35	36.2	4.5	16.3	1.7	43.3	5.6	2.33	0.83
	**72**	15.8	3.1	41.7	3.3	6.98	0.78	24.9	1.9	9.13	0.47
	**144**	37.80	0.10	22.1	1.3	3.23	0.32	17.6	3.1	19.3	2.2

**C-2045**	**24**	2.40	0.44	27.7	2.0	12.97	0.67	53.5	2.7	3.5	1.2
	**72**	22.4	6.3	29.6	7.7	7.05	0.33	26.1	1.3	15.7	2.2
	**144**	46.3	6.4	21.6	1.4	4.87	0.85	10.5	1.3	16.8	6.2
**QD$_{green}$-C-2045**	**24**	2.70	0.92	34.0	3.7	15.6	2.4	44.4	8.4	4.8	1.4
	**72**	7.7	1.3	52.0	6.9	6.47	0.47	23.6	3.6	10.2	3.2
	**144**	43.2	3.5	26.6	5.9	4.07	0.60	10.83	0.64	15.3	4.2
**QD$_{red}$-C-2045**	**24**	2.23	0.57	37.4	1.9	17.1	2.9	39.3	3.5	3.8	1.2
	**72**	9.4	1.9	46.1	3.0	7.0	1.1	23.3	3.6	10.0	1.7
	**144**	32.3	7.0	30.5	4.2	3.23	0.58	15.1	4.3	18.8	6.9

Table S1 c. Analysis of cell cycle by flow cytometry in MRC-5 cells. Data shows percentage of cells treated with QDs, UAs and QDs-UAs hybrids at IC$_{80}$ value for the time indicated in the sub-G1, G1, S, G2/M, and Poli (polyploid cells) phases of the cell cycle. Data represented the averages of three independent experiments.

**Compound**	**Phases of the cell cycle**									
	**sub-G1**	**SD**	**G1**	**SD**	**S**	**SD**	**G2/M**	**SD**	**Poli**	**SD**
**Control**	1.00	0.17	87.1	1.7	1.50	0.52	6.90	0.36	3.6	1.7
**QD$_{green}$**	0.950	0.071	87.40	0.26	0.67	0.21	7.0	1.4	4.3	2.0
**QD$_{red}$**	1.50	0.44	88.6	1.4	1.27	0.25	7.5	1.2	2.07	0.68
**C-2028**	4.3	1.9	64.3	4.3	3.5	1.1	25.3	2.9	6.7	3.0
**QD$_{green}$-C-2028**	3.47	0.25	71.8	3.0	1.70	0.56	18.6	2.2	3.80	0.28
**QD$_{red}$-C-2028**	2.60	0.32	73.9	5.0	1.45	0.26	19.2	2.7	7.6	5.8
**C-2045**	3.2	1.1	64.0	5.2	3.3	1.5	24.8	3.7	4.7	1.6
**QD$_{green}$-C-2045**	2.8	1.8	70.3	3.2	2.00	0.20	21.7	1.5	3.83	0.90
**QD$_{red}$-C-2045**	5.2	1.4	66.0	4.6	2.63	0.83	22.3	3.1	5.5	3.4

Table S2 d. Analysis of cell cycle by flow cytometry in CCD 841 CoN cells. Data shows percentage of cells treated with QDs, UAs and QDs-UAs hybrids at IC$_{80}$ value for the time indicated in the sub-G1, G1, S, G2/M, and Poli (polyploid cells) phases of the cell cycle. Data represented the averages of three independent experiments.

**Compound**	**Phases of the cell cycle**									
	**sub-G1**	**SD**	**G1**	**SD**	**S**	**SD**	**G2/M**	**SD**	**Poli**	**SD**
**Control**	1.27	0.45	81.0	3.0	1.90	0.20	13.9	3.1	1.97	0.72
**QD$_{green}$**	0.90	0.44	82.5	5.6	1.40	0.20	13.7	3.4	1.5	1.9
**QD$_{red}$**	1.33	0.67	80.5	6.0	1.63	0.25	14.2	3.8	2.3	1.9
**C-2028**	3.5	1.3	53.5	3.5	3.48	0.95	29.5	2.4	12.8	2.9
**QD$_{green}$-C-2028**	1.33	0.81	71.4	4.4	1.43	0.31	21.3	1.6	4.5	1.5
**QD$_{red}$-C-2028**	2.60	0.46	67.4	2.8	3.77	0.71	22.8	1.9	2.80	0.62
**C-2045**	3.6	1.5	61.5	6.7	3.1	1.0	25.7	3.2	6.6	2.1
**QD$_{green}$-C-2045**	1.6	1.0	72.9	3.4	1.80	0.26	19.6	1.3	3.1	1.0
**QD$_{red}$-C-2045**	2.4	1.5	72.2	3.4	1.93	0.40	19.9	1.7	3.33	0.64

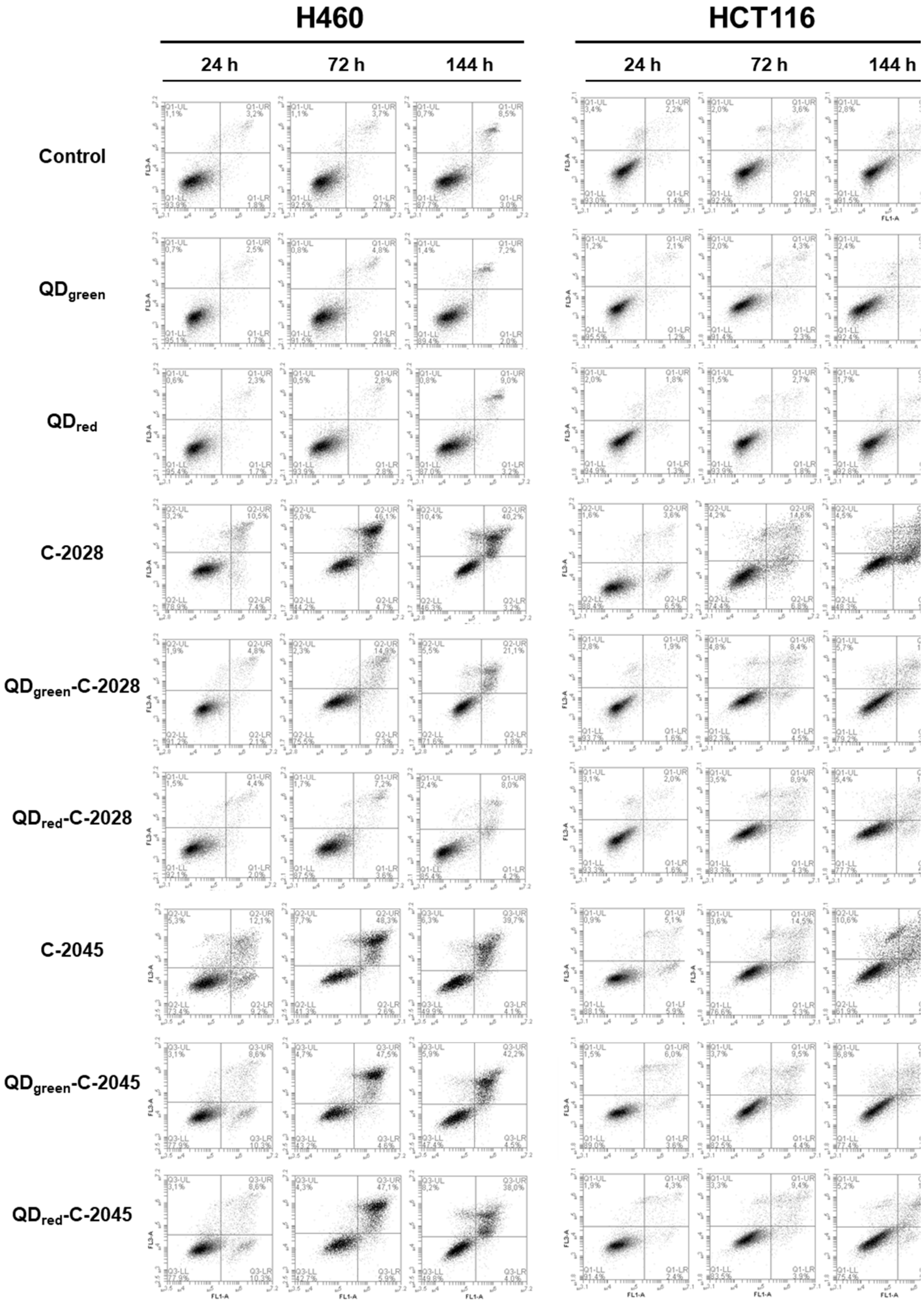

Figure S4. Flow cytometry analysis of phosphatidylserine externalization by Annexin V/propidium iodide (PI) assay. Representative histograms of cancer H460 and HCT116 cells following treatment with QDs, UAs and QDs-UAs hybrids for the time indicated. Data represented the histograms of three independent experiments. Bottom left quadrant represents live cells (Annexin V negative, PI negative); bottom right quadrant - early apoptotic cells (Annexin V positive, PI negative); top right quadrant – late apoptotic cells

(Annexin V positive, PI positive); top left quadrant - primary necrotic cells (Annexin V negative, PI positive).

Table S2 a. Flow cytometry analysis of phosphatidylserine externalization by Annexin V/propidium iodide (PI) assay in H460 cells. Percentage of cells treated with QDs, UAs and QDs-UAs hybrids at IC_{80} value. Data represented the averages of three independent experiments. A-/PI- (Annexin V negative, PI negative) represents live cells; A+/PI- (Annexin V positive, PI negative) - early apoptotic cells; A+/PI+ (Annexin V positive, PI positive) – late apoptotic cells; A-/PI+ (Annexin V negative, PI positive) - primary necrotic cells.

Compound		Percentage of cell population [%]							
		A-/PI-		A+/PI-		A+/PI+		A-/PI+	
	[h]	mean	SD	mean	SD	mean	SD	mean	SD
Control	24	91.1	2.3	1.17	0.57	5.1	1.6	2.6	1.2
	72	91.60	0.69	3.0	1.0	4.43	0.65	1.0	0.6
	144	86.2	1.5	3.07	0.21	9.7	1.2	1.0	0.3
QD_{green}	24	93.0	2.3	2.3	1.3	3.7	1.2	1.03	0.67
	72	91.5	2.2	2.90	0.10	4.7	1.4	0.93	0.65
	144	88.8	3.1	2.4	1.4	7.5	2.3	1.37	0.51
QD_{red}	24	92.3	2.7	2.27	0.90	4.0	1.5	1.4	1.3
	72	88.8	1.2	3.3	1.4	6.3	1.7	1.7	1.0
	144	88.3	2.3	2.83	0.64	7.8	1.9	1.00	0.10
C-2028	24	77.9	2.5	9.0	1.6	10.6	3.1	2.5	1.1
	72	41.3	3.3	4.4	1.0	47.8	3.7	6.6	1.6
	144	42.5	8.8	4.3	1.0	44.5	7.2	8.1	2.3
QD_{green}-C-2028	24	90.5	2.0	3.2	0.8	4.8	1.6	1.57	0.49
	72	78.3	3.3	4.4	1.6	14.0	2.6	3.20	0.82
	144	70.70	0.75	3.30	0.46	21.8	1.3	4.23	0.65
QD_{red}-C-2028	24	91.6	2.6	2.5	1.4	4.0	1.2	1.9	1.0
	72	86.9	5.1	3.0	1.6	8.0	3.1	2.13	0.55
	144	84.5	1.9	2.63	0.67	10.4	1.5	2.5	1.3
C-2045	24	74.2	4.3	12.1	4.2	10.97	0.90	2.7	1.8
	72	42.4	6.7	5.4	1.7	45.4	8.5	6.8	2.0
	144	45.0	8.5	5.8	4.1	40.7	3.9	8.57	0.65
QD_{green}-C-2045	24	77.5	5.3	12.4	4.1	7.7	1.1	2.37	0.91
	72	38.4	7.0	5.9	1.5	49.4	4.5	6.2	3.8
	144	42.1	5.9	3.9	1.1	45.3	4.8	8.6	2.1
QD_{red}-C-2045	24	83.3	3.1	7.5	1.7	6.57	0.47	2.6	1.2
	72	46.5	3.1	6.1	1.7	41.3	4.2	6.0	2.8
	144	50.2	1.5	4.10	0.78	38.0	1.3	7.7	1.3

Table S2 b. Flow cytometry analysis of phosphatidylserine externalization by Annexin V/propidium iodide (PI) assay in HCT116 cells. Percentage of cells treated with QDs, UAs and QDs-UAs hybrids at IC_{80} value. Data represented the averages of three independent experiments. A-/PI- (Annexin V negative, PI negative) represents live cells; A+/PI- (Annexin V positive, PI negative) - early apoptotic cells; A+/PI+ (Annexin V positive, PI positive) – late apoptotic cells; A-/PI+ (Annexin V negative, PI positive) - primary necrotic cells.

Compound		Percentage of cell population [%]							
		A-/PI-		A+/PI-		A+/PI+		A-/PI+	
	[h]	mean	SD	mean	SD	mean	SD	mean	SD
Control	24	92.00	0.79	2.13	0.21	3.67	0.90	2.27	0.83
	72	91.03	0.45	2.77	0.40	3.83	0.15	2.37	0.51
	144	90.7	1.8	2.13	0.72	4.4	1.3	2.77	0.60
QD_{green}	24	93.6	1.2	1.57	0.21	3.3	1.0	1.47	0.31
	72	92.6	1.3	2.20	0.26	3.67	0.55	1.50	0.50
	144	89.9	1.2	2.43	0.21	4.10	0.66	3.6	2.1
QD_{red}	24	91.8	2.2	2.10	0.26	4.5	2.2	1.60	0.35
	72	91.5	1.7	3.00	0.62	3.8	0.7	1.7	1.0
	144	90.80	0.61	2.43	0.67	4.2	0.5	2.50	0.56
C-2028	24	84.4	3.8	6.30	0.53	7.4	4.4	1.93	0.76
	72	70.2	5.7	8.1	1.9	17.5	6.3	4.2	1.2
	144	54.1	8.5	14.1	3.3	26.8	7.4	5.07	0.55
QD_{green}-C-2028	24	90.5	2.7	2.53	0.40	4.8	2.7	2.13	0.76
	72	82.8	2.1	5.3	1.0	7.5	1.7	4.30	0.66
	144	77.13	0.90	7.5	1.1	10.30	0.46	5.1	1.7
QD_{red}-C-2028	24	91.3	1.0	2.20	0.20	4.4	2.0	2.1	1.1
	72	84.8	4.7	5.0	1.6	7.3	3.2	2.87	0.50
	144	78.9	2.8	8.0	2.4	9.2	1.1	3.67	0.81
C-2045	24	86.9	6.3	4.5	1.8	7.0	4.6	1.53	0.29
	72	74.9	7.4	8.6	4.0	12.8	5.0	3.7	1.5
	144	46.5	7.4	14.9	5.8	33.1	3.0	5.53	0.40
QD_{green}-C-2045	24	86.4	4.7	3.97	0.87	8.0	4.8	1.6	1.1
	72	80.6	3.3	6.5	1.6	9.5	1.3	3.40	0.44
	144	73.6	5.4	7.1	2.3	13.5	3.3	5.77	0.21
QD_{red}-C-2045	24	89.2	2.2	3.23	0.29	5.8	2.8	1.80	0.30
	72	80.4	4.9	6.0	1.5	10.0	3.3	3.63	0.38
	144	74.2	3.1	7.9	1.3	12.30	0.85	5.5	1.9

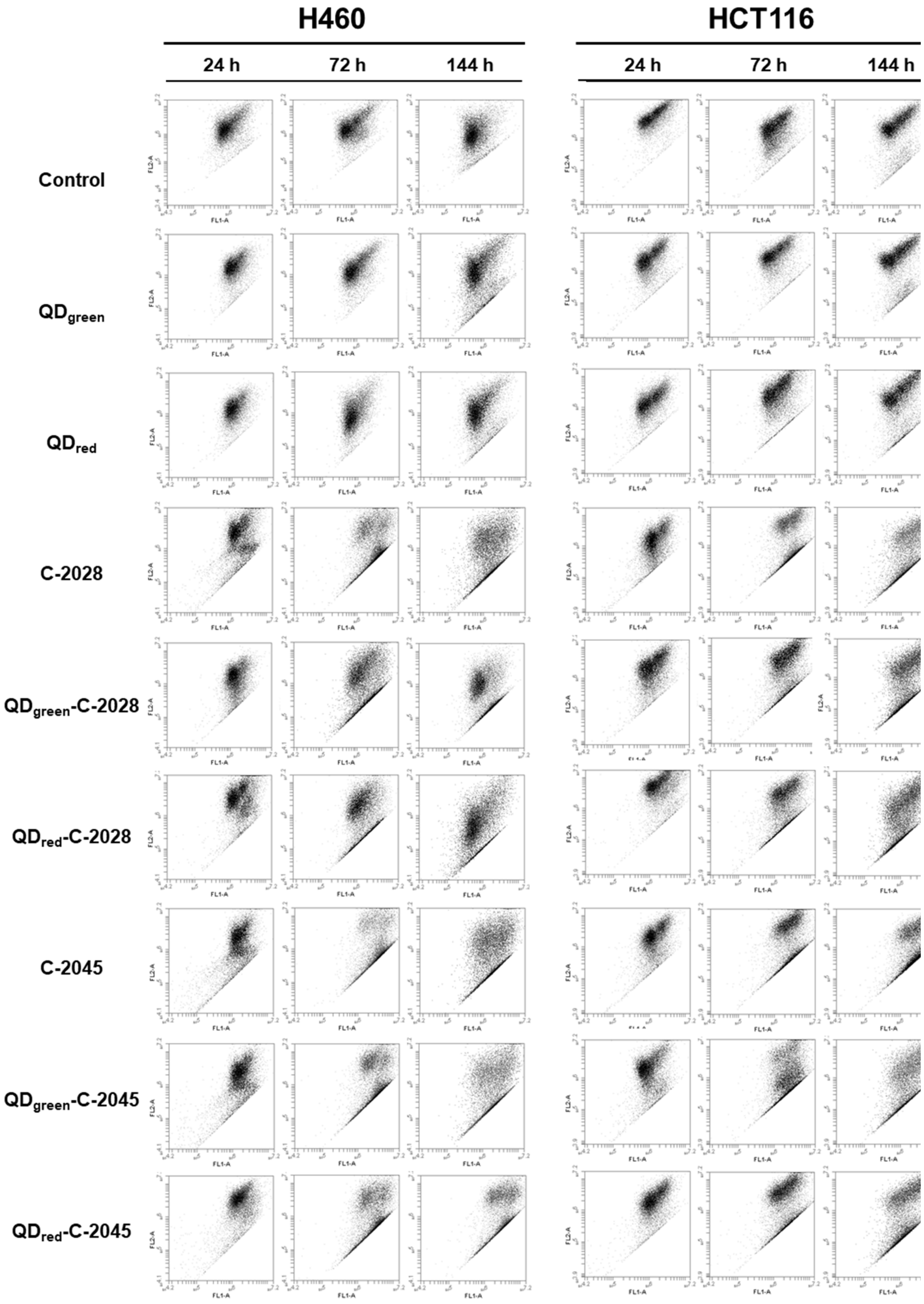

Figure S5. Analysis of the changes in mitochondrial membrane potential ($\Delta\Psi_m$). Representative histograms of cancer H460 and HCT116 cells following treatment with QDs, UAs and QDs-UAs hybrids for the time indicated. $\Delta\Psi_m$ was measured by flow cytometry using JC-1 dye. Data represented the histograms of three independent experiments.

Table S3 a. Cytometric analysis of changes in mitochondrial transmembrane potential (ΔΨm) in H460 cells. Cell were treated with QDs, UAs and QDs-UAs hybrids at IC_{80} value for the time indicated. Data shows percentage of cells with decreased mitochondrial transmembrane potential (ΔΨm) (green fluorescence). Data represented the averages of three independent experiments.

Compound	24 h		72 h		144 h	
	Mean	SD	Mean	SD	Mean	SD
**Control**	2.77	0.70	2.1	1.1	8.6	2.4
**QD_{green}**	3.47	0.81	2.5	1.1	11.1	1.9
**QD_{red}**	2.77	0.50	3.1	1.7	7.6	2.1
**C-2028**	11.6	2.5	41.0	5.4	45.8	3.3
**QD_{green}-C-2028**	5.6	2.4	29.07	0.93	27.8	3.5
**QD_{red}-C-2028**	5.1	1.8	20.4	4.7	16.7	2.0
**C-2045**	15.2	3.0	61.4	3.9	43.4	3.1
**QD_{green}-C-2045**	12.3	2.6	47.5	1.6	54.1	5.0
**QD_{red}-C-2045**	5.73	0.58	51.53	0.93	46.7	4.0

Table S3 b. Cytometric analysis of changes in mitochondrial transmembrane potential (ΔΨm) in HCT116 cells. Cell were treated with QDs, UAs and QDs-UAs hybrids at IC_{80} value for the time indicated. Data shows percentage of cells with decreased mitochondrial transmembrane potential (ΔΨm) (green fluorescence). Data represented the averages of three independent experiments

Compound	24 h		72 h		144 h	
	Mean	SD	Mean	SD	Mean	SD
**Control**	1.63	0.61	3.2	1.2	7.4	1.2
**QD_{green}**	2.07	0.84	2.87	0.80	10.7	2.8
**QD_{red}**	3.1	1.1	5.6	1.9	9.30	0.70
**C-2028**	7.27	0.67	40.7	2.4	55.3	2.9
**QD_{green}-C-2028**	4.6	1.8	17.37	0.91	36.2	2.6
**QD_{red}-C-2028**	4.6	1.5	23.6	4.3	33.4	1.1
**C-2045**	6.7	2.7	30.7	2.3	39.3	3.5
**QD_{green}-C-2045**	7.4	2.9	30.1	3.4	53.2	2.2
**QD_{red}-C-2045**	4.93	0.71	23.0	2.0	44.0	5.2

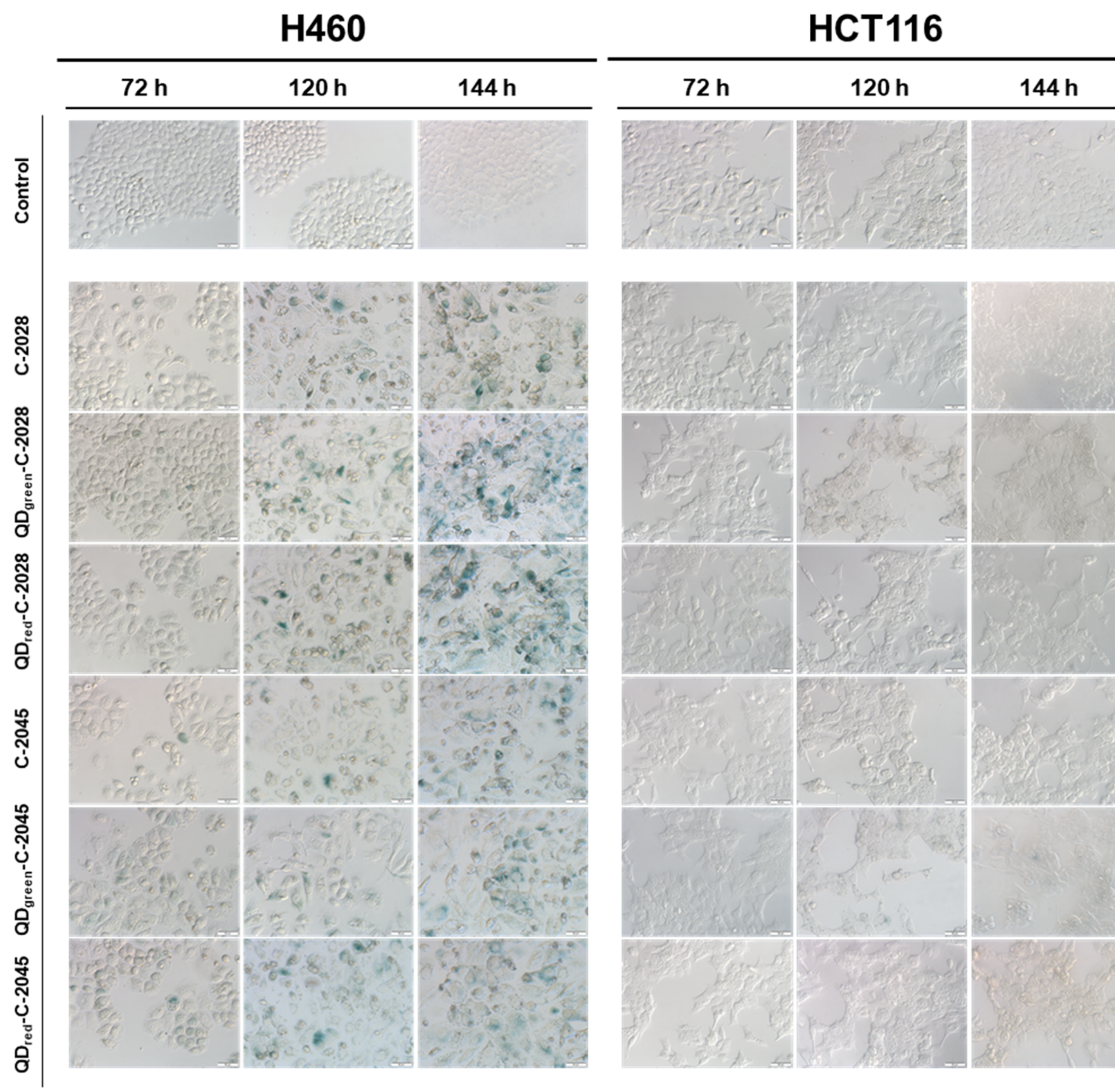

Figure S6. Cellular senescence of H460 and HCT116 cancer cells following treatment with UAs and QDs-UAs hybrids for the time indicated. Senescence-associated β-galactosidase activities were assessed by X-gal staining using light microscope. Data represented the images of three independent experiments. The scale bar 50 μm.
